# Recent Advances and Challenges in Long Wavelength Sensitive Cationic Photoinitiating Systems

**DOI:** 10.3390/polym15112524

**Published:** 2023-05-30

**Authors:** Liping Zhang, Lun Li, Ying Chen, Junyi Pi, Ren Liu, Yi Zhu

**Affiliations:** 1 Key Laboratory of Synthetic and Biological Colloids, Ministry of Education, School of Chemical and Material Engineering, Jiangnan University, Wuxi 214122, China; 2 International Research Center for Photoresponsive Molecules and Materials, Jiangnan University, Wuxi 214122, China

**Keywords:** photopolymerization, cationic photoinitiating systems, onium salt, UV/visible LED lights, long wavelength

## Abstract

With the advantages offered by cationic photopolymerization (CP) such as broad wavelength activation, tolerance to oxygen, low shrinkage and the possibility of “dark cure”, it has attracted extensive attention in photoresist, deep curing and other fields in recent years. The applied photoinitiating systems (PIS) play a crucial role as they can affect the speed and type of the polymerization and properties of the materials formed. In the past few decades, much effort has been invested into developing cationic photoinitiating systems (CPISs) that can be activated at long wavelengths and overcome technical problems and challenges faced. In this article, the latest developments in the long wavelength sensitive CPIS under ultraviolet (UV)/visible light-emitting diodes (LED) lights are reviewed. The objective is, furthermore, to show differences as well as parallels between different PIS and future perspectives.

## 1. Introduction

In the past decades, with the advantages of rapid reaction, low energy consumption, operation at room temperature and solvent-free or less solvent formulation, photopolymerization technology has become the frontier field for macromolecular synthesis and has been successfully applied in various fields [1], such as coatings [2,3,4], 3D printing [5,6,7], food packaging [8,9,10], dental materials [11,12] and photoresist [13]. Nowadays, photopolymerization has become one of the most preferred technologies for the fabrication of complex macromolecular structures in a fast and highly accurate manner.

Photoinitiating systems (PIS) play a very important role in the process of photopolymerization, which not only determines the type of photopolymerization, but also affects the speed of the polymerization and the final performance of the polymers formed [14]. Depending on the type of initiating species, photopolymerizations can proceed by free radical photopolymerization (FRP), cationic photopolymerization (CP) or even anionic mechanisms.

Compared with the FRP mode, CP has many advantages such as the possibility of post-curing, no oxygen inhibition [15], wider application range and so on [16]. Additionally, the cationically polymerizable monomers have low toxicity, irritation and volume shrinkage [17,18,19]. These excellent properties make it widely used in industry.

**Scheme 1:**
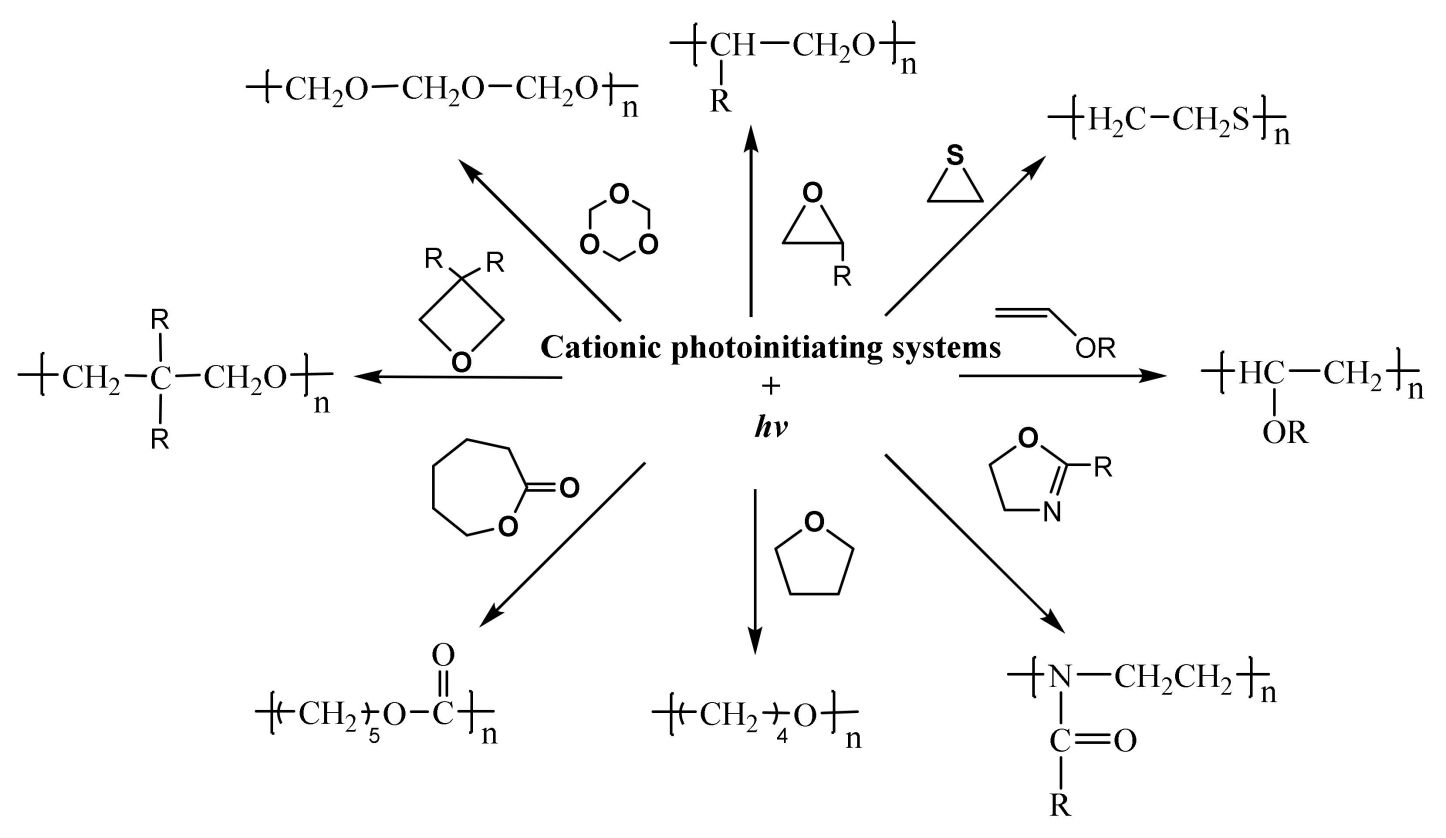
Types of monomers for cationic photopolymerization [18].

**Scheme 2:**
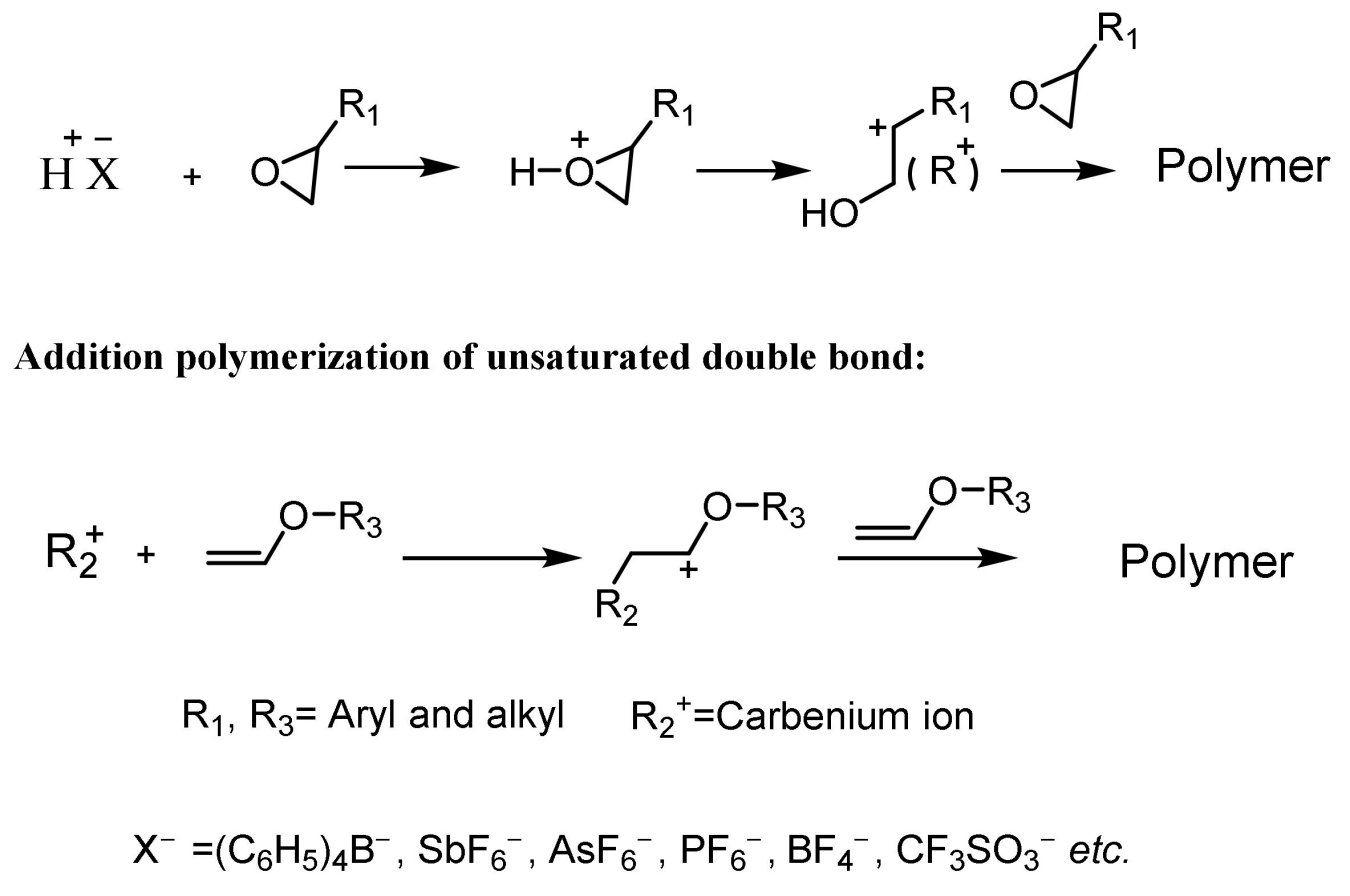
Mechanism of cationic photopolymerization [18].

Certain vinyl and epoxy monomers, such as alkyl vinyl ethers and industrially important cyclic ethers, lactones and cycloacetals, respectively, cannot be polymerized by a radical mechanism, but readily polymerize using an ionic initiator. Scheme 1 presents the list of cationically polymerizable monomers and their corresponding polymers. Typical general reactions for the CP of vinyl and epoxy monomers proceeding via electrophilic addition and ring opening processes are depicted in Scheme 2 [18].

The light absorption range of the most widely used commercial CPISs, namely diaryliodonium salts and triarylsulfonium salts, is below 350 nm. Thus, high-energy light sources are needed for the excitation of photoinitiators (PIs) [20]. However, ozone, excess heat and light pollution produced during the photopolymerization process by high-energy light sources such as a mercury lamp, are the major concerns in industrial use [21]. To date, various approaches to extend absorption characteristics of CPIS to longer wavelengths to provide a lower energy pathway have been reported. In general, such aim can be realized in two different ways: (i) by introducing highly conjugated groups into PI structure (single-component PI) or (ii) using additives that can undergo energy or electron transfer reactions with the PI (multi-component PIS).

In this review, both strategies are briefly summarized, and recent developments are highlighted and portrayed how these techniques can enlarge the range of long wavelength CPISs suitable for industrial applications.

## 2. Single-Component Cationic Photoinitiators

Single-component cationic photoinitiators (CPIs), also known as photoacid generators, absorb photons and in the excited state generate Bronsted acid directly. Single-component CPIs generally possess two components: cationic and anionic, which play different roles in CP. As a photosensitive group, cation part absorbs photons, then undergo energy level transition, and also determines the molar extinction coefficient, spectral absorption range, quantum yield and thermal stability of the PI [22,23]. The anionic part also affects the polymerization efficiency as termination occurs with highly nucleophilic counter anions. Therefore, the anion has to be sufficiently nonnucleophilic to prevent the termination of a growing chain–cation combination. In general, the common anion reactivity is increased in the order (C_6_H_5_)_4_B^−^ > SbF_6_^−^ > AsF_6_^−^ > PF_6_^−^ > BF_4_^−^ > CF_3_SO_3_^−^ [24,25]. In the following section, single-component PIs are discussed according to their ionic or nonionic structures.

### 2.1. Ionic Photoinitiators

The ionic PIs have a hetero atom salt structure with cationic centers on the heteroatoms coupled with non-nucleophilic counter anions.

#### 2.1.1. Aryl Diazonium Salts

**Scheme 3:**
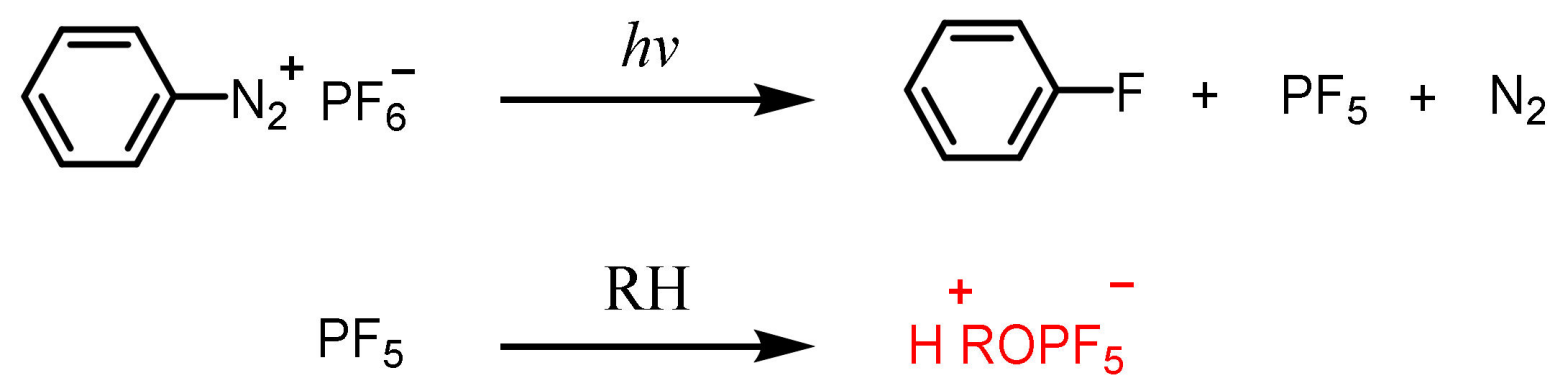
Photolysis mechanism of aryl diazonium salts [26]. Red color represent the active species that can initiate polymerization.

As one of the earliest reported CPIs, aryl diazonium salts can release N_2_ and Lewis acids, and form Bronsted acids by reacting with proton donors (RH) under the irradiation of light source. As is well known, Lewis acids and Bronsted acids formed in the photolysis process can be used as active species to initiate CP [26] (Scheme 3). Despite their high initiation efficiency, aryl diazonium salts have some disadvantages, such as the release of nitrogen from the initiating system during photolysis, which can have a large impact on the properties of the final materials. Meanwhile, the poor thermal stability further limits their industrial application of aryl diazonium salts [27].

#### 2.1.2. Iodonium Salts

**Scheme 4:**
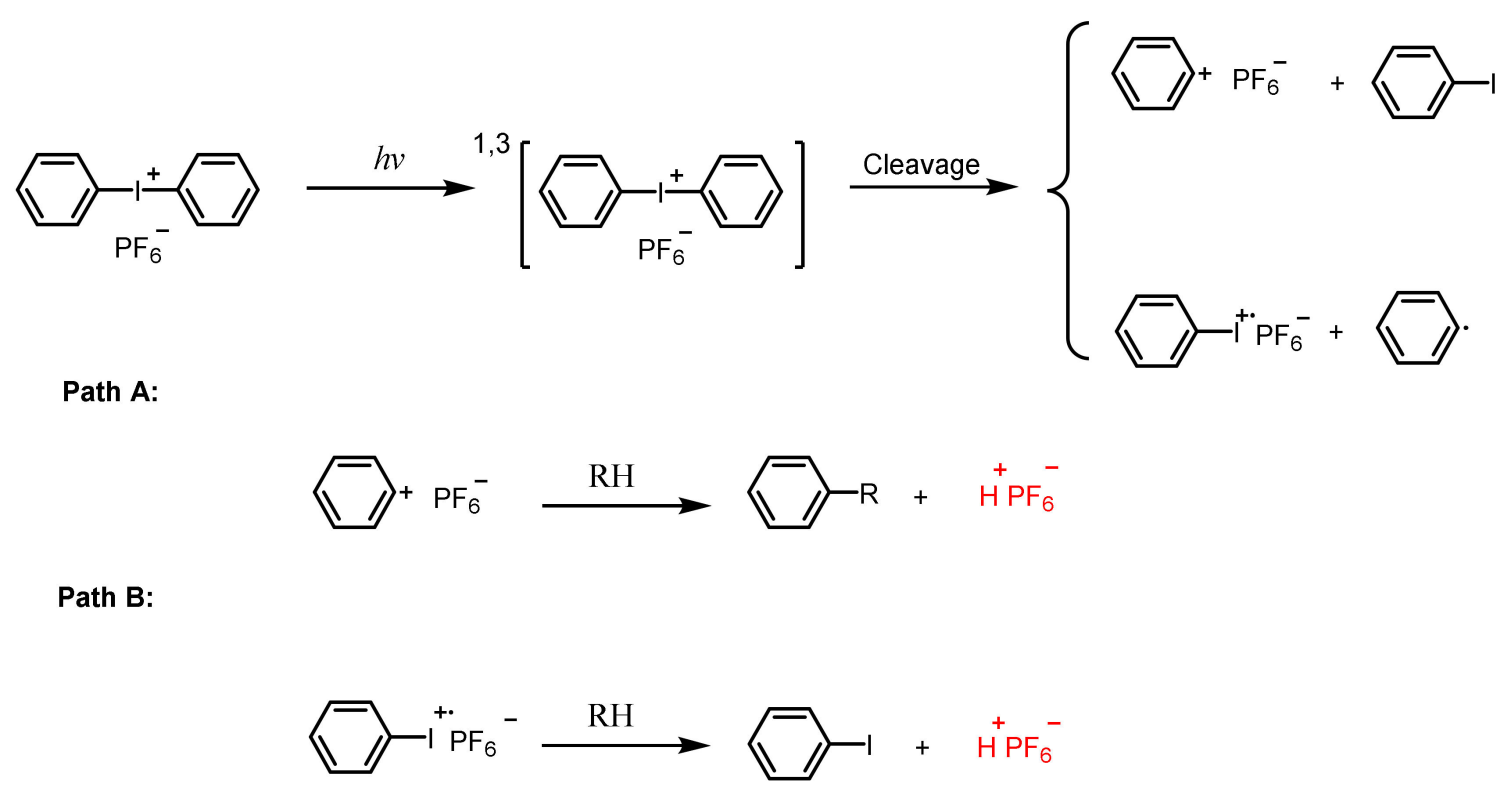
Photolysis mechanism of iodonium salt [23]. Red color represent the active species that can initiate polymerization.

Diaryl iodonium salts as CPIs were first reported by Crivello et al. [23] in the 1970s. With the advantages of excellent photoinitiated activity, easy synthesis, no gas generation and excellent stability, diaryl iodonium salts have been well developed and widely used in CP. The photolysis mechanism of diaryl iodonium salt is depicted in Scheme 4.

However, the absorption wavelength of diaryl iodonium salts is usually below 300 nm, which greatly limits their applications. There are two common ways to extend their spectral sensitivity to longer wavelengths. The first approach concerns the extension of conjugation by introducing additional chromophoric groups to the structure. H. Schroder et al. [28] replaced the aryl group of diphenyl iodonium salt with 9-fluorenone, resulting in the extension of the conjugation. The new compound had two weak absorption bands at 380 nm and 394 nm with molar extinction coefficients of 430 and 400 L mol^−1^ cm^−1^, respectively.

Following this study, a series of iodonium salts with different chromophores have been synthesized and used as PIs. For example, J. Lalevée et al. [29] reported a novel iodonium salt containing naphthalimide (naphthalimide-Ph-I^+^-Ph) as a single-component PI, which causes the red-shift of the absorption wavelength of the initiator, allowing polymerization to occur at longer and safer wavelengths (i.e., violet-emitting diodes at 365, 385 and 395 nm). This PI can effectively initiate the polymerization of various formulations (methacrylates, epoxides, vinyl ethers). Due to the excellent photophysical and chemical properties of coumarin, it has also been used as a chromophore introduced into iodonium salts. Four new coumarin-based PIs are described by Ortyl et al. [15] as a single-component PI for the CP of epoxides, vinyl ethers, oxiranes and glycerol-based monomers, as well as hybrid formulations under visible light. The hybrid polymerizations (HP) applied were successfully used to construct interpenetrating polymer networks (IPN) polymer materials in the field of 3D printing. Optical microscopy experiments show that structures can be printed with good resolution in these hybrid resins by a 3D stereolithography process. In 2021, Orty et al. [30] introduced a series of novel one-component iodonium salt photoinitiators based on benzylidene scaffolds, which contain double bonds and dialkylamino groups, and were synthesized in one step through a classical aldol condensation reaction. Novel benzylidene iodonium salts can photoinitiate the polymerization of vinyl ether and epoxy monomers under LED@ 365 nm and LED@ 405 nm illumination. The investigated compounds can simultaneously initiate and monitor the polymerization process based on changes in fluorescence during photocuring. Formulations prior to photopolymerization show no fluorescence; during photopolymerization, the fluorescence is “turned on”. This phenomenon can be used to monitor photopolymerization in an “online” manner. The structures of some common and typical iodonium salts are shown in Table 1.

Beside the commonly used iodonium salts, dialyl chloronium salts and dialyl bromonium salts can also be used as CPIs [33]. These salts have a similar skeleton structure, UV absorption spectrum and photolysis mechanism to iodonium salts. It is worth noting that the change of the central halogen atom results in a lower difference, the synthesis yields of dialyl chloronium salts and dialyl bromonium salts are relatively low, and the materials obtained by using these PIs are dark colored, which limits their practical applications.

#### 2.1.3. Sulfonium Salts

##### Triarylsulfonium Salts

Following the first report on triarylsulfonium salts in 1970s, Crivello and his collaborators have made outstanding contributions to the development of highly efficient PIs [34,35,36,37]. Among the various onium salts, triarylsulfonium salts have excellent photosensitivity, photoacid quantum yields (between 0.6 and 0.9), and excellent thermal stability (the decomposition temperature exceeds 120 °C.) [35].

With the development of LED light technology and the improvement of environmental requirements, triarylsulfonium salts also face the challenges of short absorption wavelength and further improve the initiating efficiency. Accordingly, a large amount of work is devoted to extending the absorption wavelength. The main strategy was to increase the number of aromatic rings and introduce a chromophore on the benzene ring, which could increase the electron delocalization of the PI, resulting in a red-shift on the absorption wavelength of the PI. At the same time, it could also increase the molar extinction coefficient of the absorption of the PI [38]. For example, the introduction of a phenylthio group to a triarylsulfonium salt results in a red-shift of the maximum absorption wavelength from 227 nm to 313 nm [39]. Three typical triaryl sulfonium salt PIs designed according to the above strategy are listed in Table 2.

**Scheme 5:**
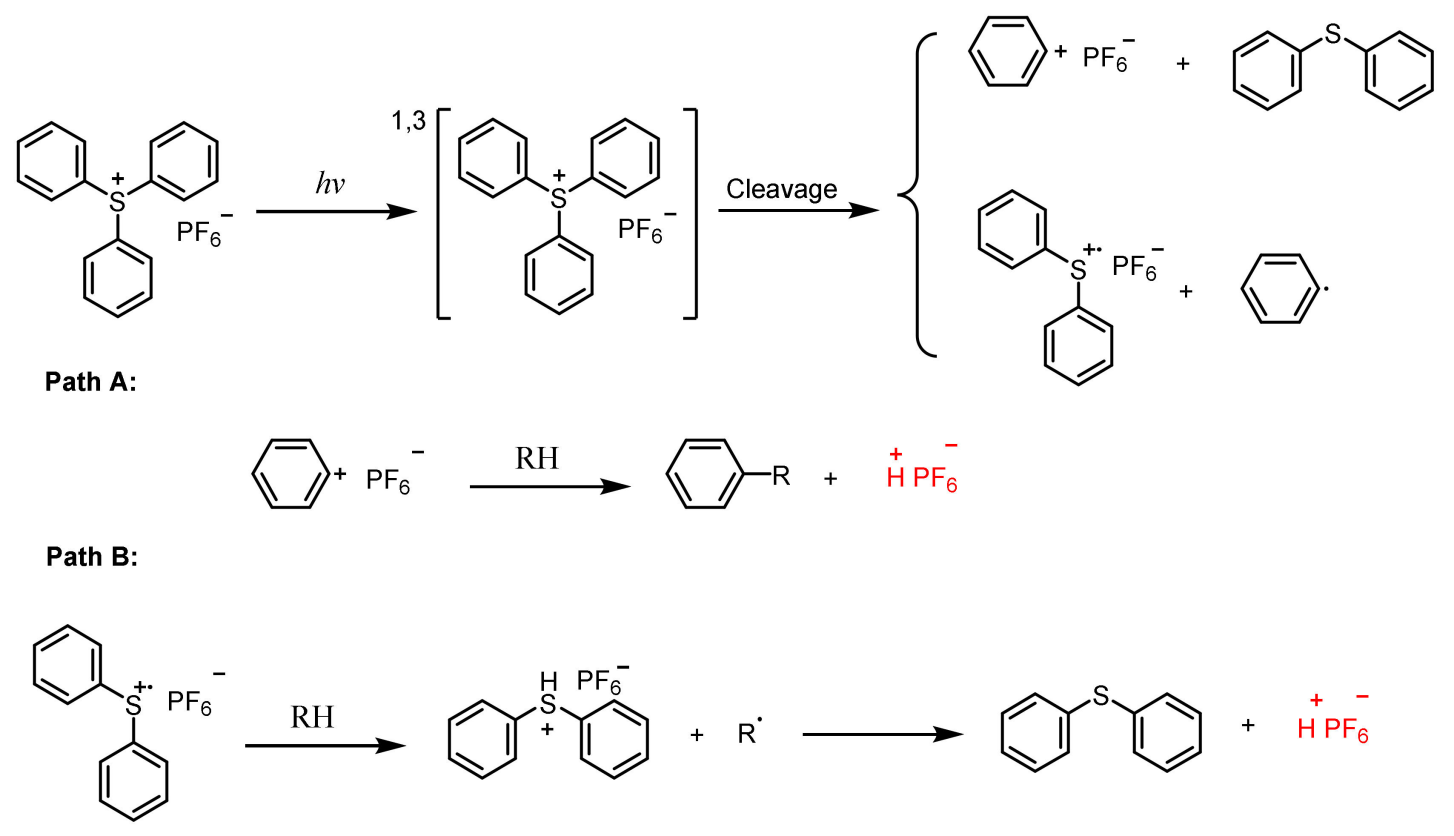
Photolysis mechanism of triarylsulfonium salt [27]. Red color represent the active species that can initiate polymerization.

Triarylsulfonium salts produce active species through two photolysis mechanisms: hetero- and homo-cleavage. Upon irradiation, the C-S bond of the salt is cleaved to generate a phenyl cation or a diarylsulfonium cation radical, which generates reactive protonic acid after the subsequent interaction with the proton donor. The overall mechanism is shown in Scheme 5 [35,40,41,42].

**Scheme 6:**
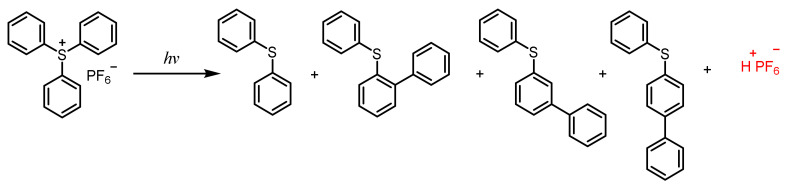
Photolysis products of triarylsulfonium salts [27]. Red color represent the active species that can initiate polymerization.

Another possible mechanism was found when the researchers analyzed the photolysis products. As photolysis products, phenyl cations and phenyl radicals can react with diphenyl sulfide to form three positionally substituted products [27] in the decreased order of ortho > meta > para positions (Scheme 6). In this process, the form of proton is still the core active species for initiating CP. These cationic photoinitiators suffer from poor solubility in epoxy resins and emit foul odors. These shortcomings greatly limit the application of photoinitiators. Based on this, Sun et al. [43] reported three new polysiloxane-modified 5-arylsulfonium salt cationic photoinitiators (1187-Si-A/B/C), whose polysiloxane-modified cations can be considered environmentally friendly. The photoinitiator was synthesized based on 9-(4-hydroxyethoxyphenyl) hexafluorophosphate (Esacure 1187) and polysiloxane. Cationic photoinitiators not only exhibit excellent solubility in epoxy resins, but also do not release odors and toxic by-products under UV irradiation.

##### Aryl-Alkylsulfonium Salts

**Scheme 7:**
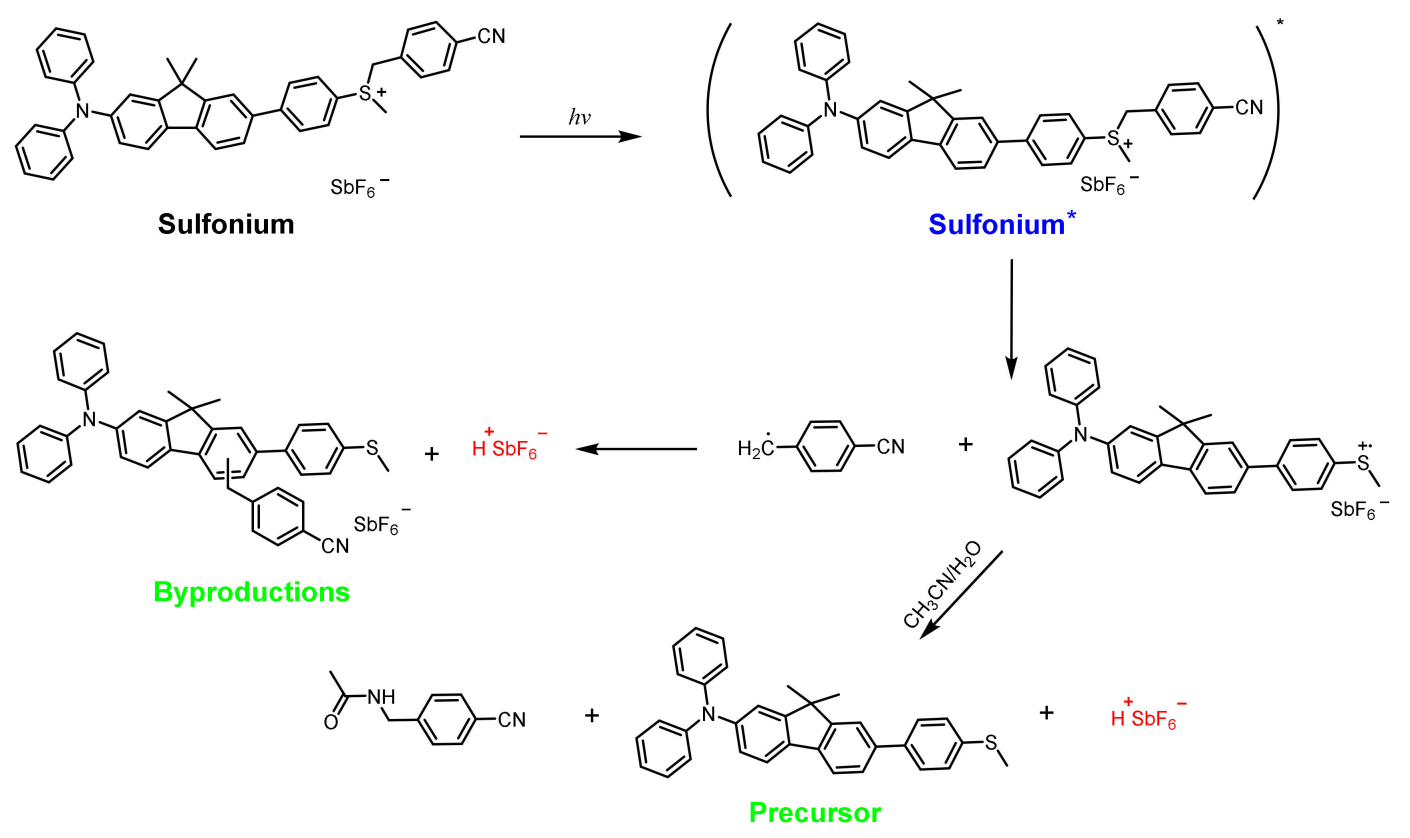
Proposed photolysis mechanism of aryl-alkylsulfonium salts [47]. Red color represent the active species that can initiate polymerization. * means that compound is in the excited state.

Trialkylsulfonium salts have poor thermal stability (thermal polymerization can occur at 50 °C) and could spontaneously initiate the polymerization of reactive monomers [27]. Fortunately, aryl-alkylsulfonium salts do not have the above problems. The photolysis mechanism of these compounds is relatively similar. Under irradiation, the sulfonium salt will undergo homo- or hetero-cleavage of the C-S bond to generate sulfur radical cations and carbon radicals. Sulfur radical cations can undergo substitution with carbon radicals or electron transfer with hydrogen donors, essentially producing Bronsted acid (Scheme 7). Jin et al., reported several strategies in this line [38,44,45,46,47,48,49]. The main strategy for designing such CPIs is to make the framework of PIs contain long conjugated structures such as D-π-A or D-π-A-D, which can not only cause a red-shift in the absorption, but also excellent acid production efficiency. In 2021, Li et al. [50] reported for the first time that a single component sulfonium salt photoinitiator combined with up-conversion nanoparticles successfully achieved near-infrared-induced cationic and free radical/cation hybrid photopolymerization. The resulting luminescent polymer material has a curing depth of more than 10 cm, good network uniformity and dual-wavelength responsiveness, which have great potential in sensing and anti-counterfeiting applications. Some typical and common aryl-alkylsulfonium salts are presented in Table 3.

##### Phenacylsulfonium Salts

**Scheme 8:**
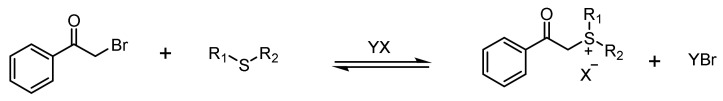
General synthetic method of phenacylsulfonium salt [52].

**Scheme 9:**
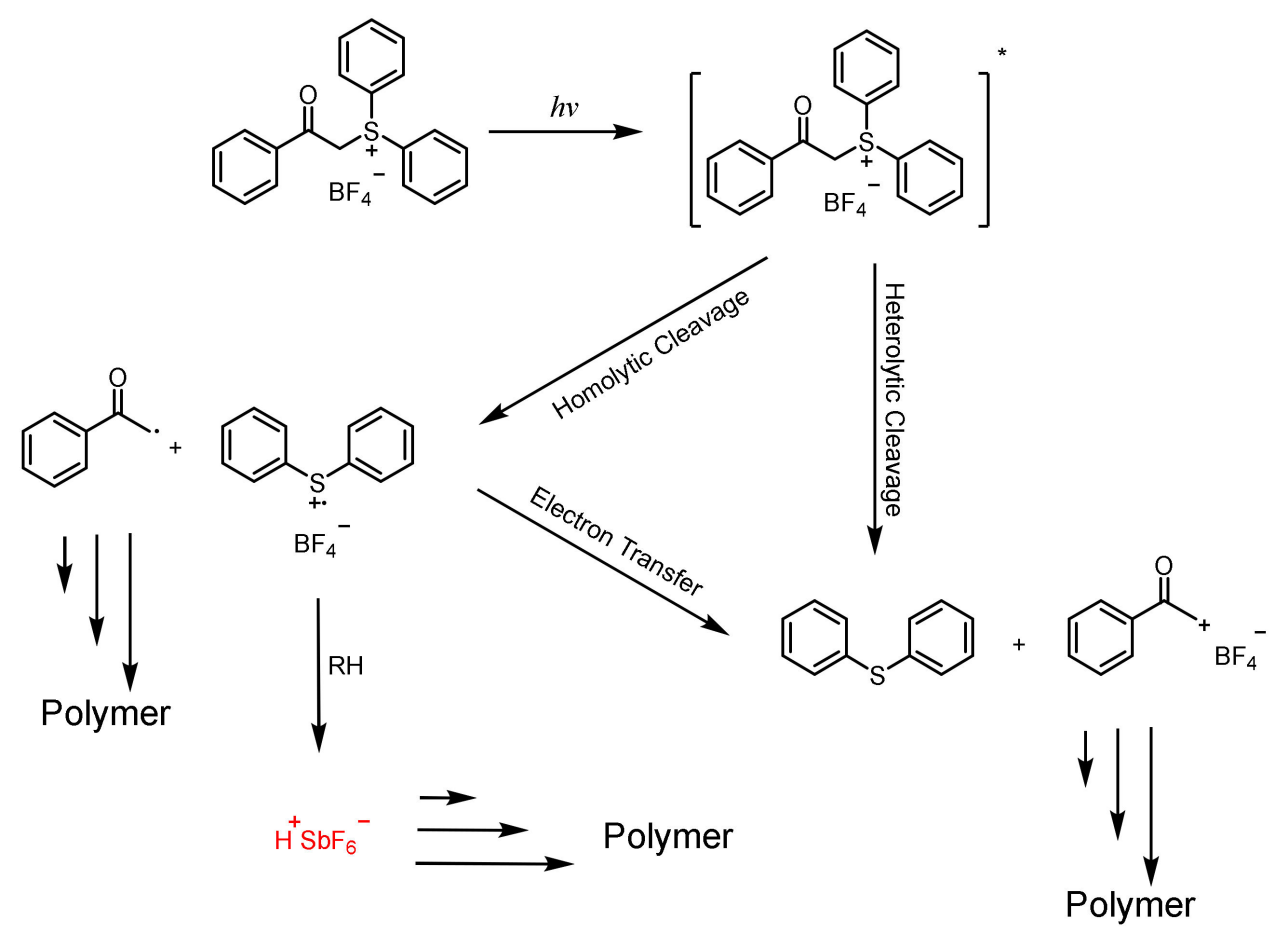
Proposed photolysis mechanism of phenacylsulfonium salts [52]. Red color represent the active species that can initiate polymerization. * means that compound is in the excited state.

Crivello et al. [51] proposed a facile method for the synthesis of dialkylphenacylsulfonium salt CPIs. Dialkylphenacylsulfonium salts can be obtained by mixing 2-bromoacetophenone and sulfide to produce bromine salt followed by anion exchange reaction (Scheme 8) [52]. In this work, it was shown that the compatibility of dialkylphenacylsulfonium salts with non-polar monomers and oligomers can be improved by changing the length of the alkyl chain. Yagci and Crivello et al. designed and synthetized a series of phenacylsulfonium salts [47,48,49,50,51]. There are two cleavage pathways of phenacylsulfonium salts under light irradiation: (i) homolysis to produce phenylacetyl radicals and sulfur radical cations; (ii) heterolysis to produce phenylacetyl cationic species. Phenylacetyl radicals are capable of initiating FRP. These free radicals can also undergo dimerization and hydrogen abstraction reactions. Sulfur radical cations can further react with hydrogen donors to generate Bronsted acids, which subsequently initiate CP [52] (Scheme 9). Liu et al. [53] reported a novel broad-wavelength absorbing phenacylsulfonium salt possessing phenacylphenothiazine chromophore that can effectively initiate CP and FRP under UV, visible and NIR irradiation. Phenacylsulfonium salts have attracted much attention in the field of photopolymerization due to their simple synthesis and adjustable properties. In 2023, based on the guidance of theoretical calculations, Liu et al. [54] designed and synthesized sulfonium salt photoinitiators based on different alkyl chains of coumarin skeleton. The resulting coumarin sulfonium salt (CSS) was used as a single-component photoinitiator for cationic photopolymerization, free radical photopolymerization and hybrid polymerization. This work clarified the effect of aliphatic chain length on photoinitiation activity. The research results provide some guidance for the design of new efficient sulfonium salt photoinitiators with controllable activity and solubility. Table 4 listed the chemical structures and absorption bands of phenacylsulfonium salts reported in the literature [51,52,55,56,57].

#### 2.1.4. Phosphonium Salts

**Scheme 10:**
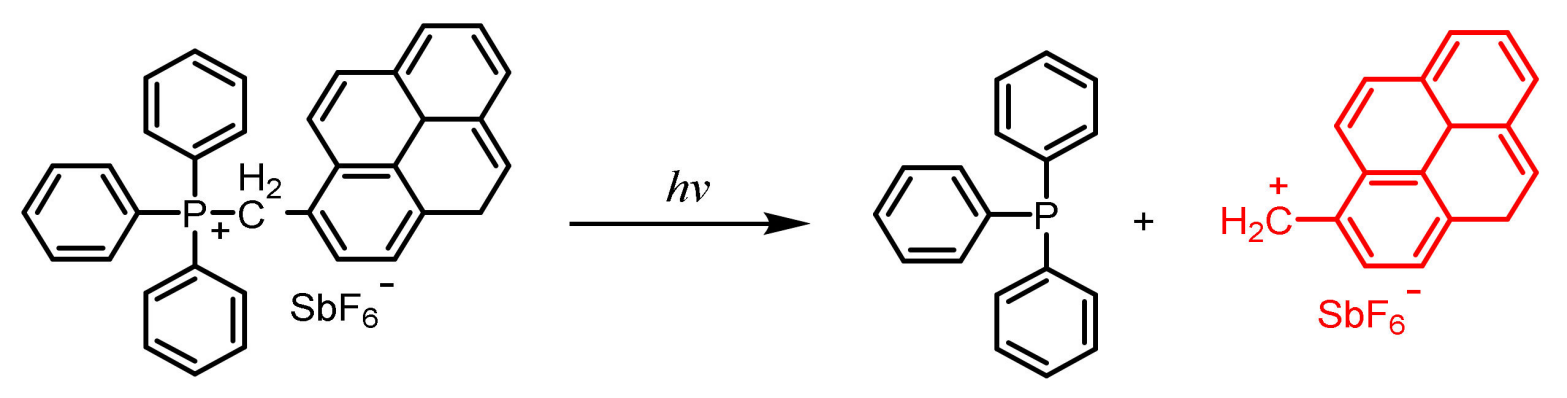
Proposed photolysis mechanism of benzoylsulfonium salts [61]. Red color represent the active species that can initiate polymerization.

Phosphonium salts can be obtained by chloro- or bromo-methylating reactions of the related aryl compounds with the corresponding phosphine-containing compounds [58,59,60]. For phosphonium salts, the active species that initiate CP are generally considered to be carbocations formed by the photochemical cleavage of the P-C bond (Scheme 10).

The phosphonium salts of pyrene methyl have good initiation ability for epoxy and vinyl monomers with almost quantitative conversion [61]. The UV-Vis and ^1^H-NMR spectral analysis of the obtained polymers provided clear evidence for the presence of aromatic end groups, confirming the involvement of pyrene methyl carbocations in the initiation process. Three common phosphonium salts used as initiators are shown in Table 5.

#### 2.1.5. Ammonium Salts

##### N-Alkoxypyridinium Salts

N-Alkoxypyridinium salts can be prepared from the corresponding pyridyl oxynitrides and can be obtained with a high yield. This type of onium salt usually produces alkaline by-products such as pyridine and isoquinoline, which can additionally consume protonic acid. The polymerization reaction may be terminated when the concentration of released base is above a certain level [62].

The UV absorption wavelength of most N-Alkoxypyridinium salts is below 300 nm, which does not match with the emission from green LED light source. The usual strategy to shift the absorption wavelength is to incorporate additional chromophores into pyridine ring. Several N-alkoxypyridinium salt PIs synthesized by this strategy are listed in Table 6 [62,63,64,65,66].

**Scheme 11:**
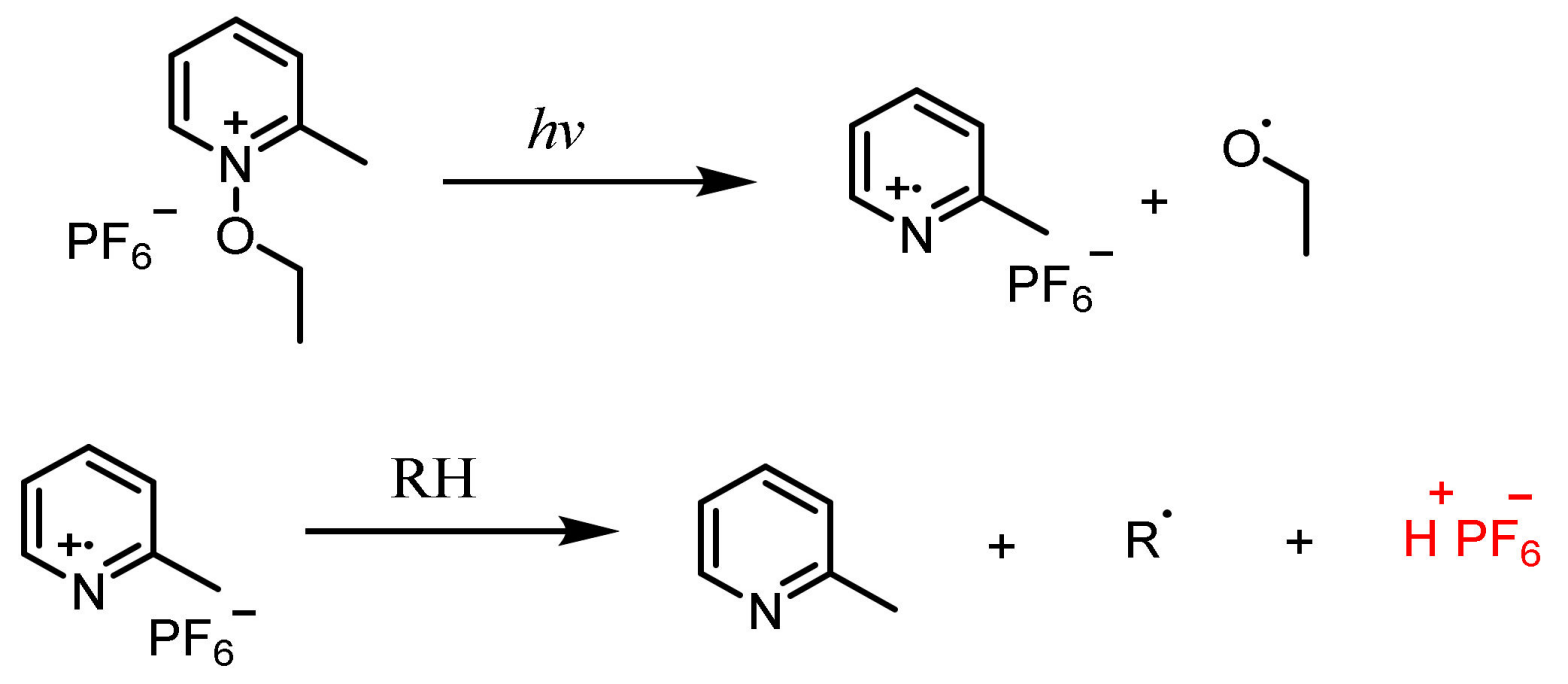
Proposed photolysis mechanism of N-Alkylpyridinium salts [61]. Red color represent the active species that can initiate polymerization.

Upon irradiation, these salts undergo hemolytic cleavage to generate pyridinium cation radicals. These radical cations abstract hydrogen and then form protonic acids to initiate a CP reaction (Scheme 11) [62,63,64,65,66].

**Table 6:** Photophysical properties of different ammonium salts.

Structure	*λ*_max_ (nm)	*ε* (L·mol^−1^·cm^−1^)	Ref.
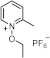	266	5925	[62]
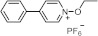	310	21,440	[62]
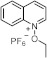	337	4220	[62]
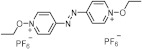	289 459	15,510 26,835	[64]

##### Phenacyl Ammonium Salts

**Scheme 12:**
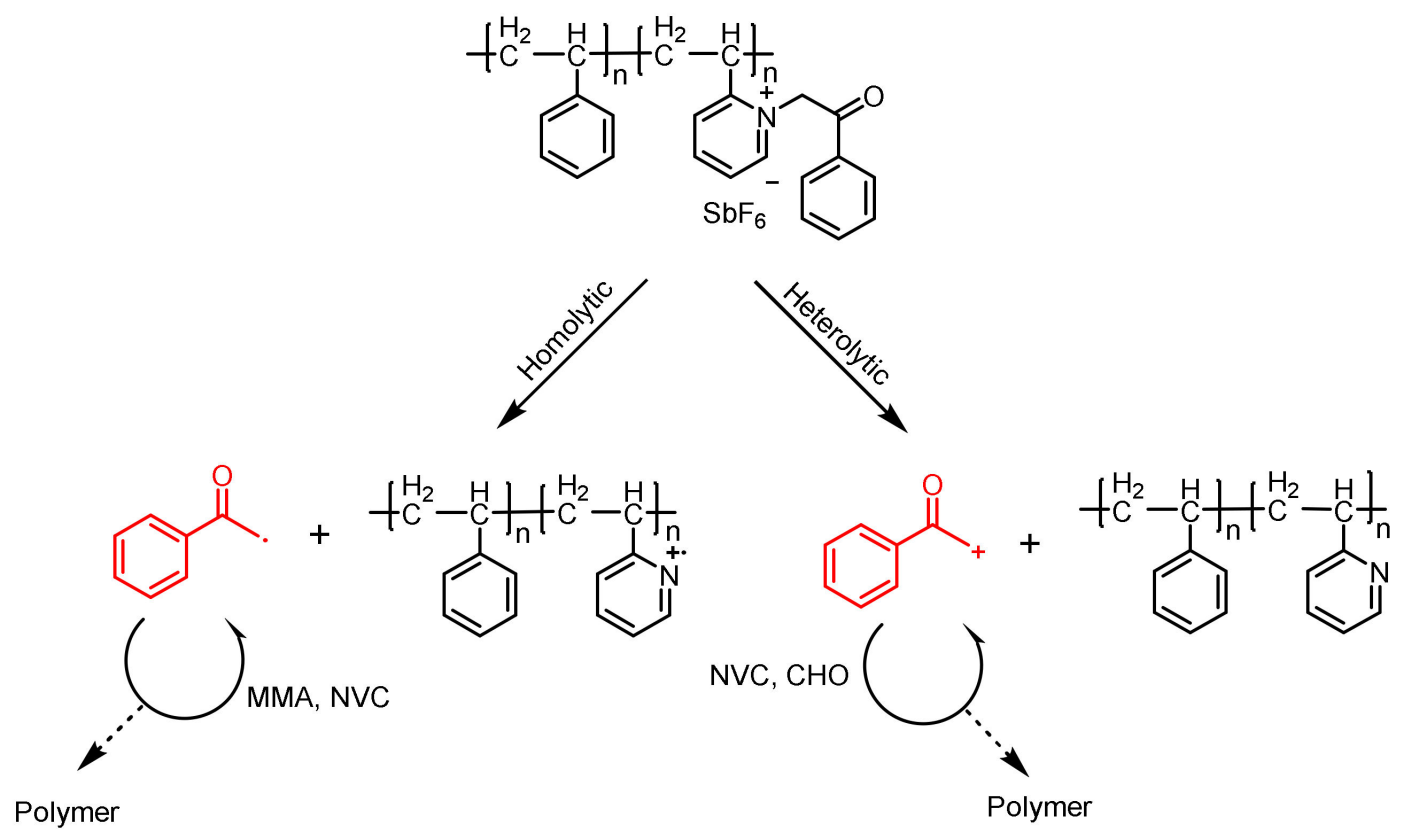
Proposed photolysis mechanism of PS-b-PVPP [63]. Red color represent the active species that can initiate polymerization.

**Figure 1:**
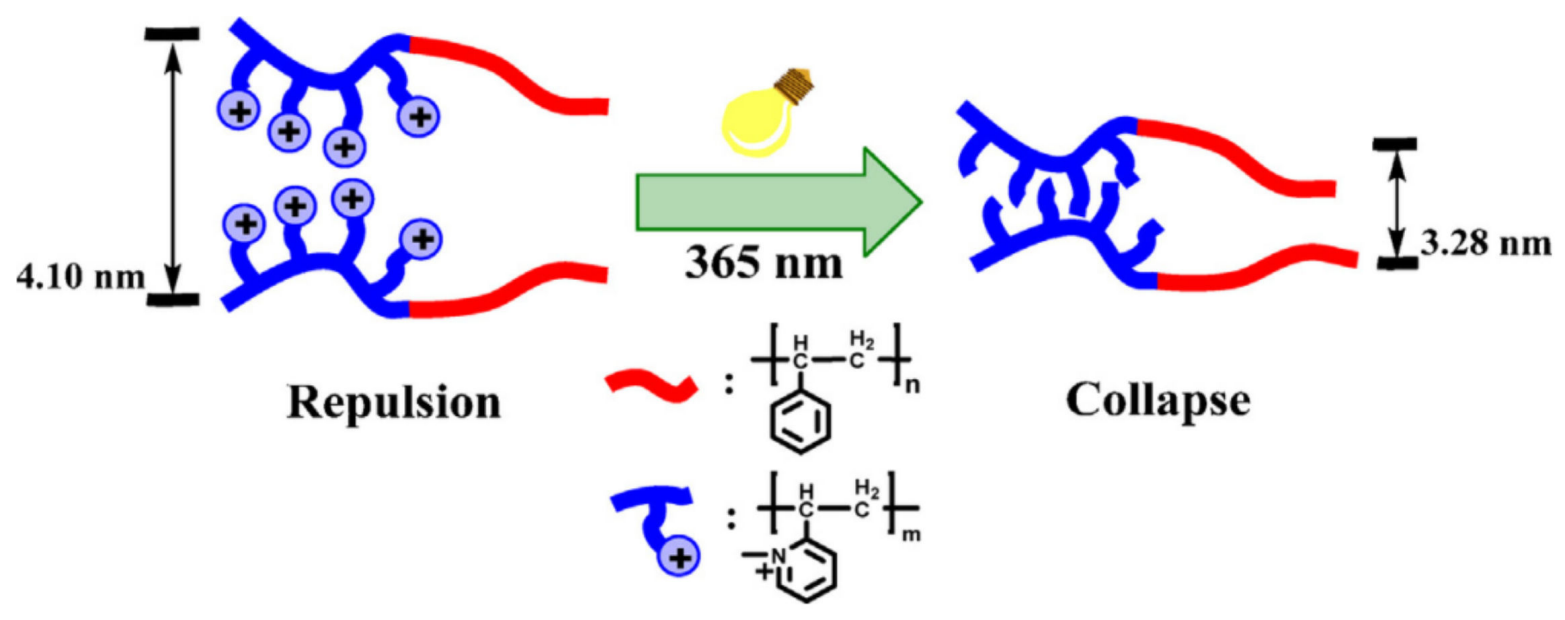
A schematic diagram of the interaction between PS-b-PVPP chains before and after 365 nm irradiation [63].

A series of phenacyl ammonium salts [67,68,69,70] as efficient PIs for FRP and CP was reported by Yagci et.al., Phenacyl ammonium salts can be obtained by S_N_1 reaction of 2-bromoacetophenone with nitrogen compounds. Several phenacyl ammonium salt photoinitiators synthesized by this strategy are listed in Table 7. In 2014, Yagci et al. [63] synthesized polystyrene-b-poly-2-vinylphenacylpyridine hexafluorophosphate (PS-b-PVPP) by phenacylation followed by anion exchange reactions of polystyrene-b-poly-2-vinylpyridine prepared by live anion polymerization. As can be seen from Scheme 12, PS-b-PVPP undergoes photolysis in two ways after absorption of photons. On one hand, benzoyl methyl radicals are produced by homolysis, which can initiate the FRP of methyl methacrylate (MMA). On the other hand, phenacylium cations formed by heterolysis initiate the CP of monomers such as epoxy cyclohexane (CHO), n-butyl vinyl ether (BVE) and N-vinyl carbazole (NVC) indicating excellent capability for HP and forming IPN. Their photochemical transition from cation to neutral state induces molecular association, leading to changes in the surface morphology of solid films and the formation of aggregates in solution. This photochemical behavior may provide new avenues for a variety of biological applications and new ways to control the surface properties and thickness of polymer films and multilayers (Figure 1). In 2023, Li et al. [71] prepared a coumarin acylaniline (CAA) onium salt by one-pot reaction. Compared with commercial iodonium salts, CAA salts exhibit excellent photoinitiation performance in cationic polymerization. Under visible light irradiation, the onium salt undergoes homolysis and cracking, followed by electron transfer and hydrogen extraction reactions to form reactive species that can initiate cationic polymerization of epoxides and vinyl monomers. After a short irradiation period, due to the non-nucleophilic nature of the counterion, polymerization also takes place in the dark. Near-infrared induced polymerization was successfully carried out on the basis of up-conversion photochemistry. CAA salt can also initiate the stepwise growth polymerization of N-ethylcarbazole (NEC) by photochemically formed aniline radical cationic oxidation monomer.

#### 2.1.6. Ferrocenium Salts

Ferroceniums salts are attractive PIs for CP [61,72,73]. These compounds can be obtained from inexpensive raw materials by simple synthetic methods. Cyclopentadiene-iron-arene salt was prepared by the ligand exchange reaction of cycloferrocene with arenes according to the method described by Nesmeyanov et al. [74]. Ferrocenium salts, such as (η6-arene) (η5-cyclopentadiene) ferric hexafluorophosphate ArFe(+)Cp, have the advantage of having larger absorption peaks in the near-UV and UV-visible light spectrum region that can be facilitated, and by changing the structure of the ligands, their absorption can be changed between long-wavelength UV and visible wavelengths.

**Scheme 13:**
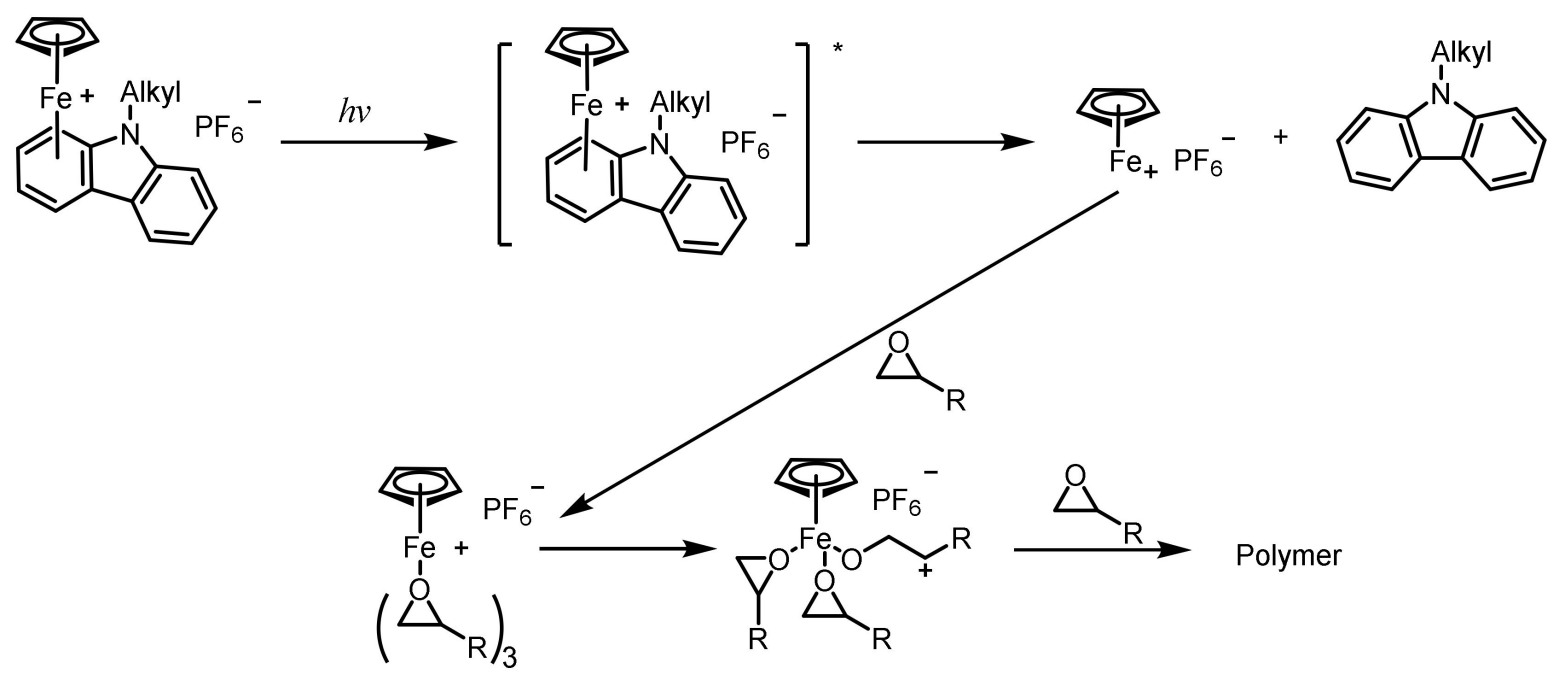
Proposed photolysis mechanism of ferrocenium salts [74]. * means that compound is in the excited state.

Ferrocene salts undergo photolysis to form iron-based Lewis acids with the loss of aromatic ligands. The latter species can coordinate with epoxy monomers, followed by ring-opening polymerization (Table 8) [75,76,77,78,79]. The photoinitiated mechanism is shown in Scheme 13.

These compounds with different substituents are single-component PIs (or key components of multi-component PIS) for the cationic ring-opening polymerization of epoxy compounds, showing activity under near-UV, blue or even green LEDs. Reviewing the recent developments in the field of CPIs, application opportunities for CP and HP based on LED irradiators may open new pathways and ferrocene salts hold great promise in this direction.

### 2.2. Nonionic Photoinitiators for Cationic Polymerization

**Scheme 14:**
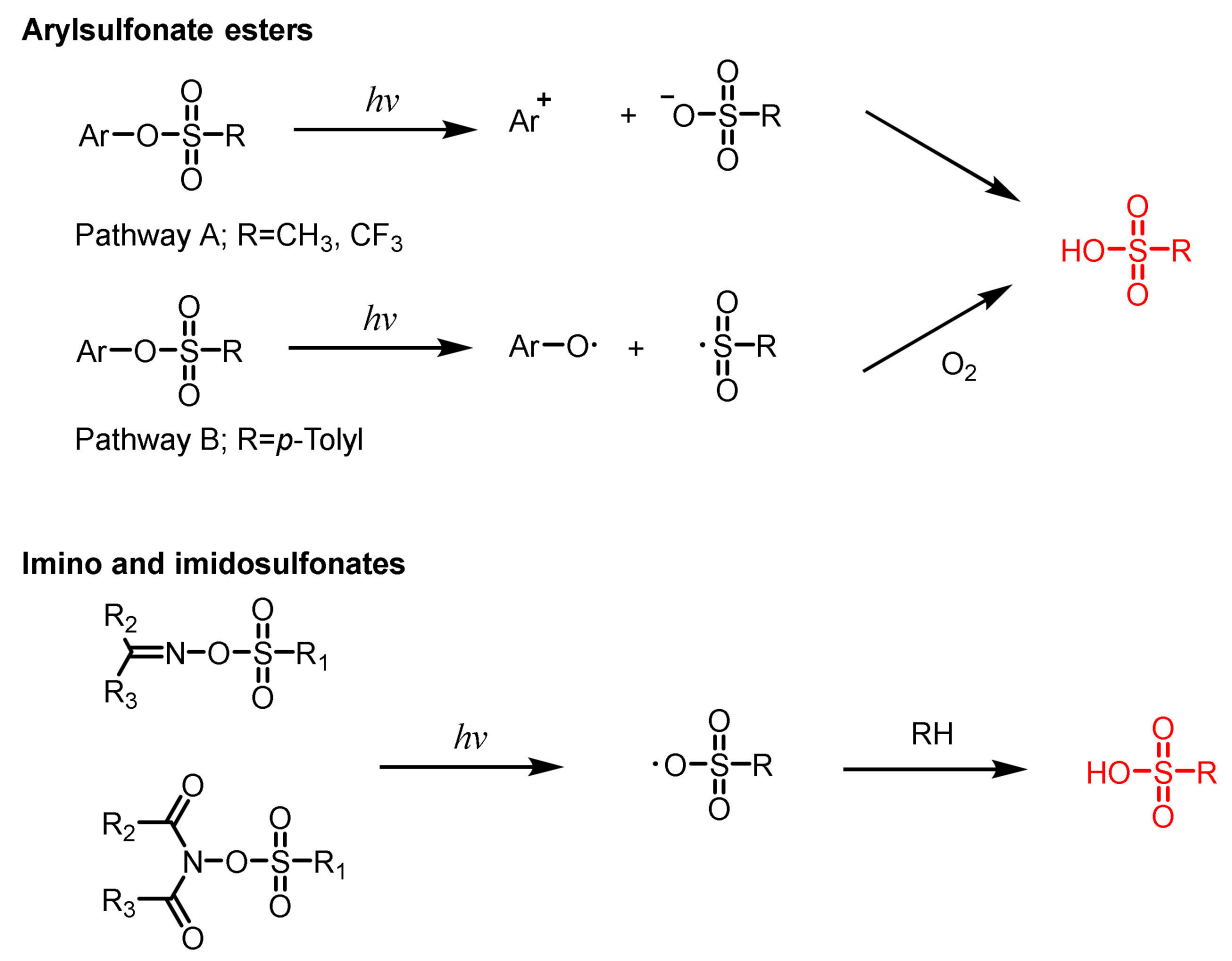
Proposed photolysis mechanism of nonionic CPs [83]. Red color represent the active species that can initiate polymerization.

Over the past few decades, various nonionic CPIs have been developed for the initiation and surface polymerization due to their better solubility in conventional solvents and resins [56,80,81,82]. Such compounds mainly include arene sulfonates, sulfamic acid esters and iminosulfonic acid esters. Under light exposure, they form structurally stable free radicals through the cleavage of C-O, S-O and N-O bonds, which can usually abstract hydrogen from the solvent, which releases acidic compounds, which are considered to be active species for initiating cationic polymerization (Scheme 14) [83]. So far, only aryl tosylates have been used in photopolymerization, capable of promoting the CP of epoxy groups in hybrid sol-gel photoresists [84].

**Scheme 15:**
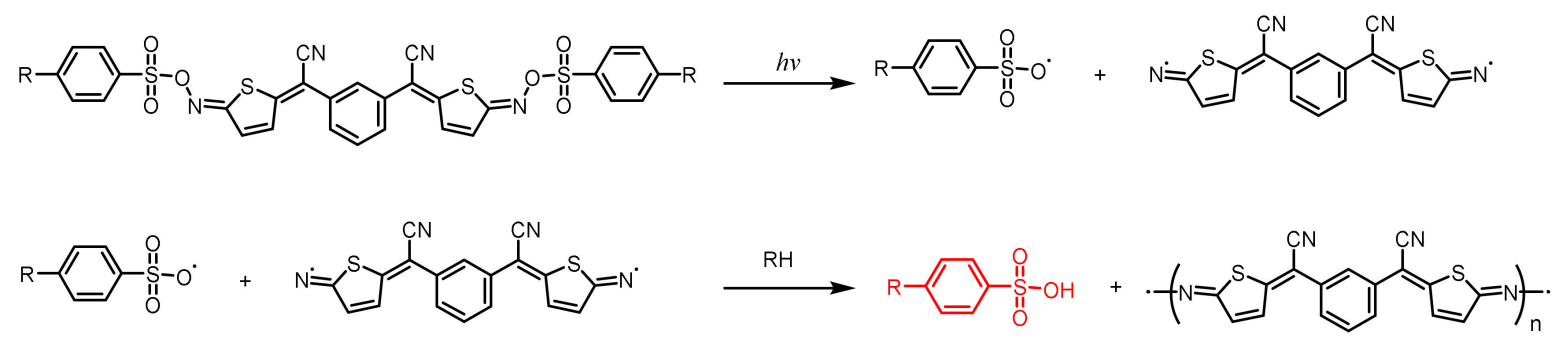
Proposed photolysis mechanism of thiophenoxime containing sulfonic acid compounds [85]. Red color represent the active species that can initiate polymerization.

Jin et al. [85] reported three novel thiophene oxime compounds containing sulfonic acid groups as non-ionic photoacid generators with large conjugated systems (Table 9). The irradiation of near-UV/visible LED (365–475 nm) leads to the cleavage of two weak N-O bonds (Scheme 15), resulting in different sulfonic acids with good quantum yields and chemical yields. These PIs are highly active and do not require any additives. They were used for CP of epoxides and vinyl ethers under low concentrations of near-UV and visible LEDs. The conversion of cyclohexene oxide can reach 99 % in the light range of 365–475 nm.

## 3. Multi-Component Cationic Photoinitiating Systems

The most widely used CPIs, such as diaryl iodonium salts and triaryl sulfonium salts, mainly absorb UV light in the range of 200–320 nm, which limits their practical applications (e.g., LEDs emitting at wavelengths greater than 360 nm). As stated above, one strategy to extend the absorption range of cationic CPIs is to incorporate chromophores into the aromatic groups of the onium salts. However, this approach requires a multi-step synthesis and purification, and is costly. It remains a challenge to obtain PIs with desirable absorption properties [86].

**Figure 2:**
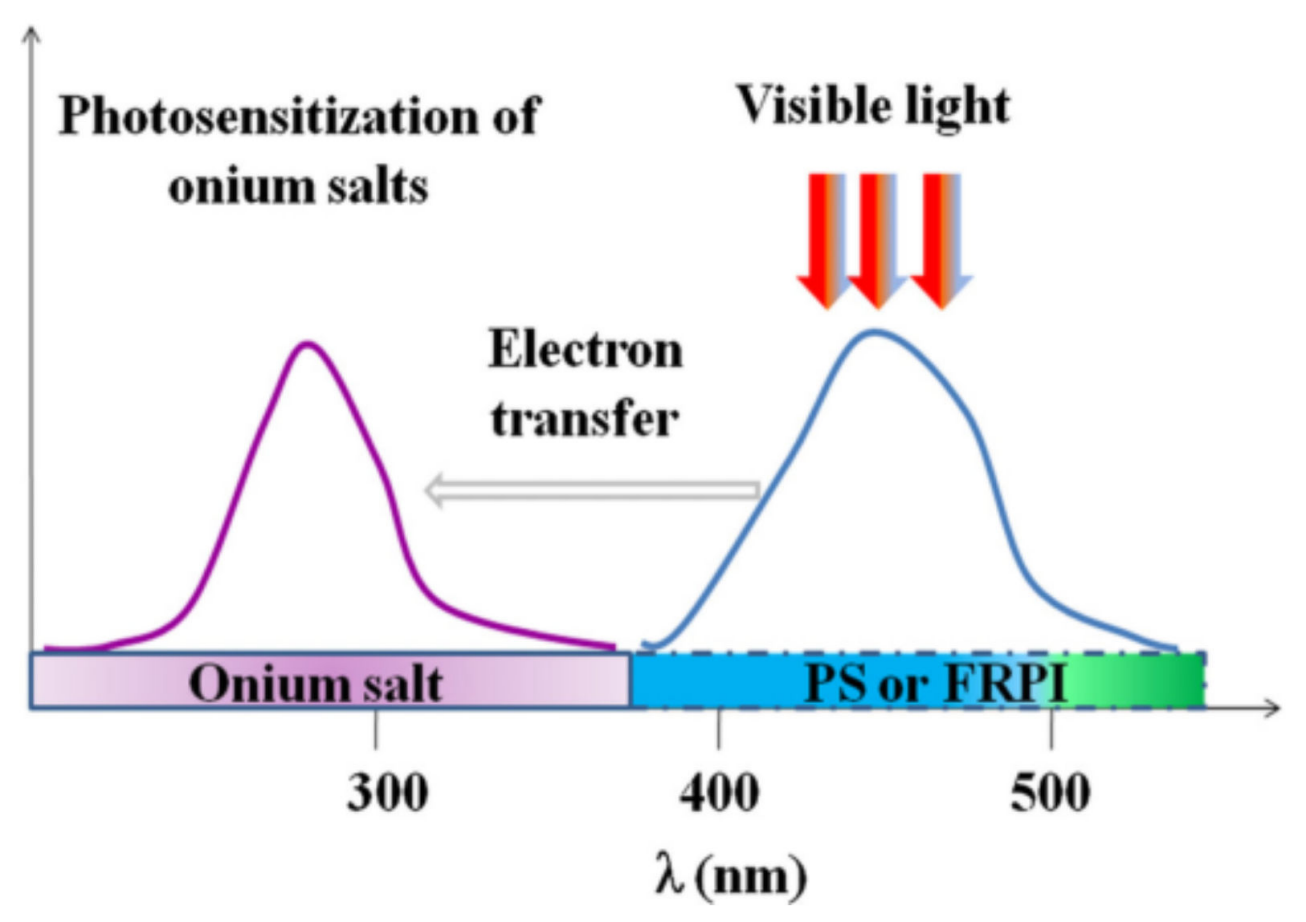
Spectral absorption range of photosensitizers and onium salts and photosensitization [86].

In order to increase the wavelength of the initiation system, the combination of onium salts and photosensitizers has been widely used to initiate CP and is considered to be a promising and effective strategy. This photosensitization generally occurs through the electron or energy transfer process between the photosensitizer or the generated free radical and the onium salt (Figure 2) [86].

### 3.1. Electron Transfer within Charge Transfer Complexes (CTC)

**Scheme 16:**
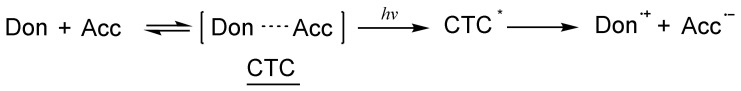
The process of CTC formation and photolysis [86]. * means that compound is in the excited state.

CTC is considered to be an efficient PIS and has been extensively studied [87,88,89,90]. The CTC is a complex composed of an electron donor molecule and an electron acceptor molecule. Among them, the electron acceptor is usually an onium salt PI, and the electron donor is an aromatic compound [91]. In the photochemical reaction process, the molecule that absorbs light energy is no longer a single molecular structure, but the CTC as a whole (Scheme 16).

**Scheme 17:**
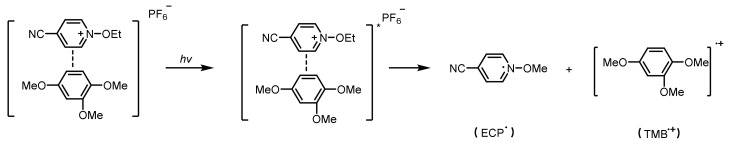
Visible light photolysis of CTC of pyridinium salt and trimethoxy benzene (TMB) [94]. * means that compound is in the excited state.

The very first CTC initiating system for CP was reported by Hizal et al. [92,93] for alkoxy pyridinium salts. In this process, CTC formed between pyridinium salt and electron donor aromatic compounds such as trimethoxy benzene (TMB) absorb the light in the visible range (Scheme 17). The radical cations of TMB or Bronsted acids formed after hydrogen abstraction successfully initiate CP of CHO.

**Figure 3:**
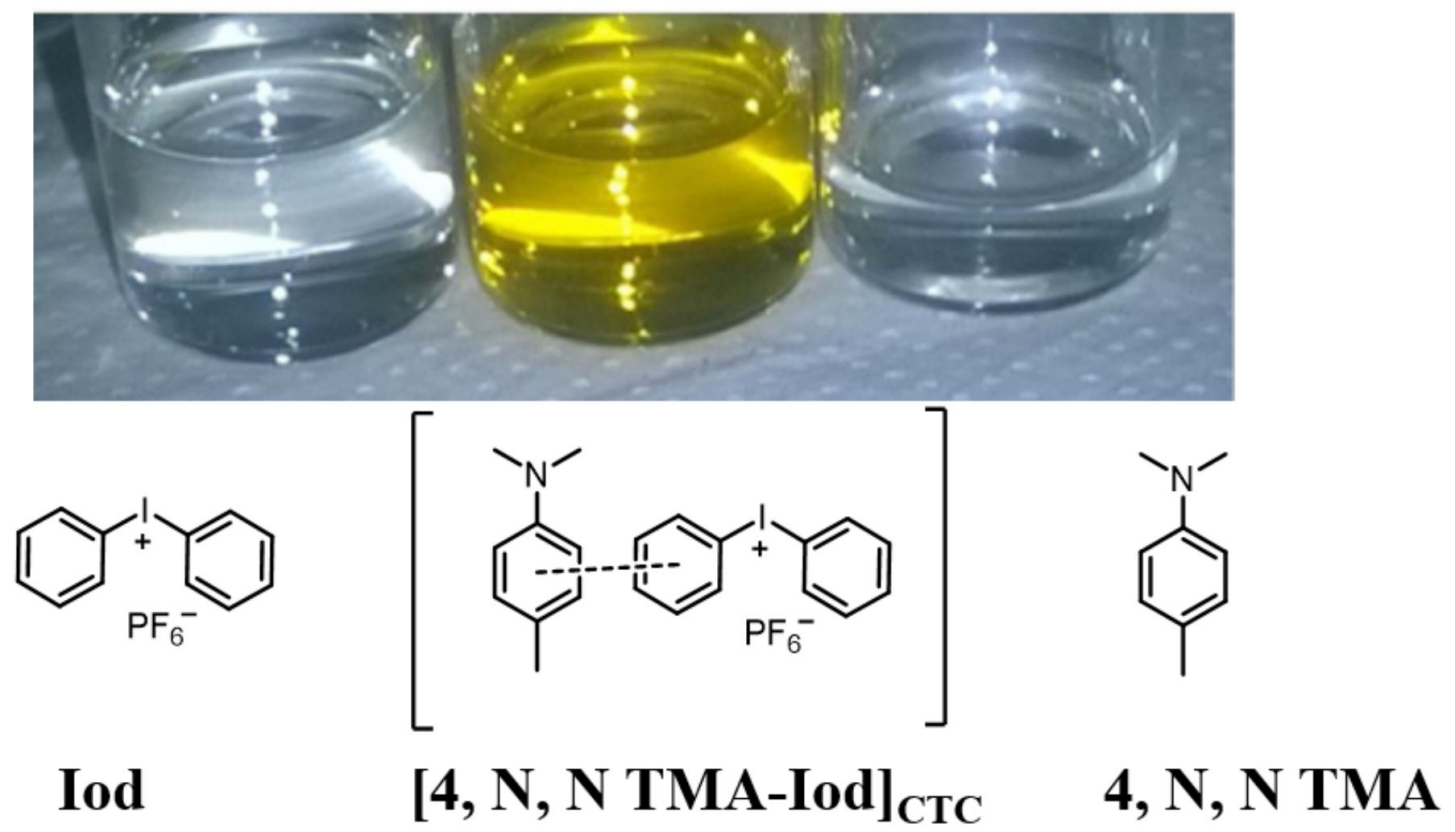
Color change of solution caused by CTC formation (0.10 M Iod (**left**)); 0.22 M 4, N, N TMA (**right**); mixture of 0.05 M Iod and 0.11 M 4, N, N TMA (**middle**)) [94].

**Figure 4:**
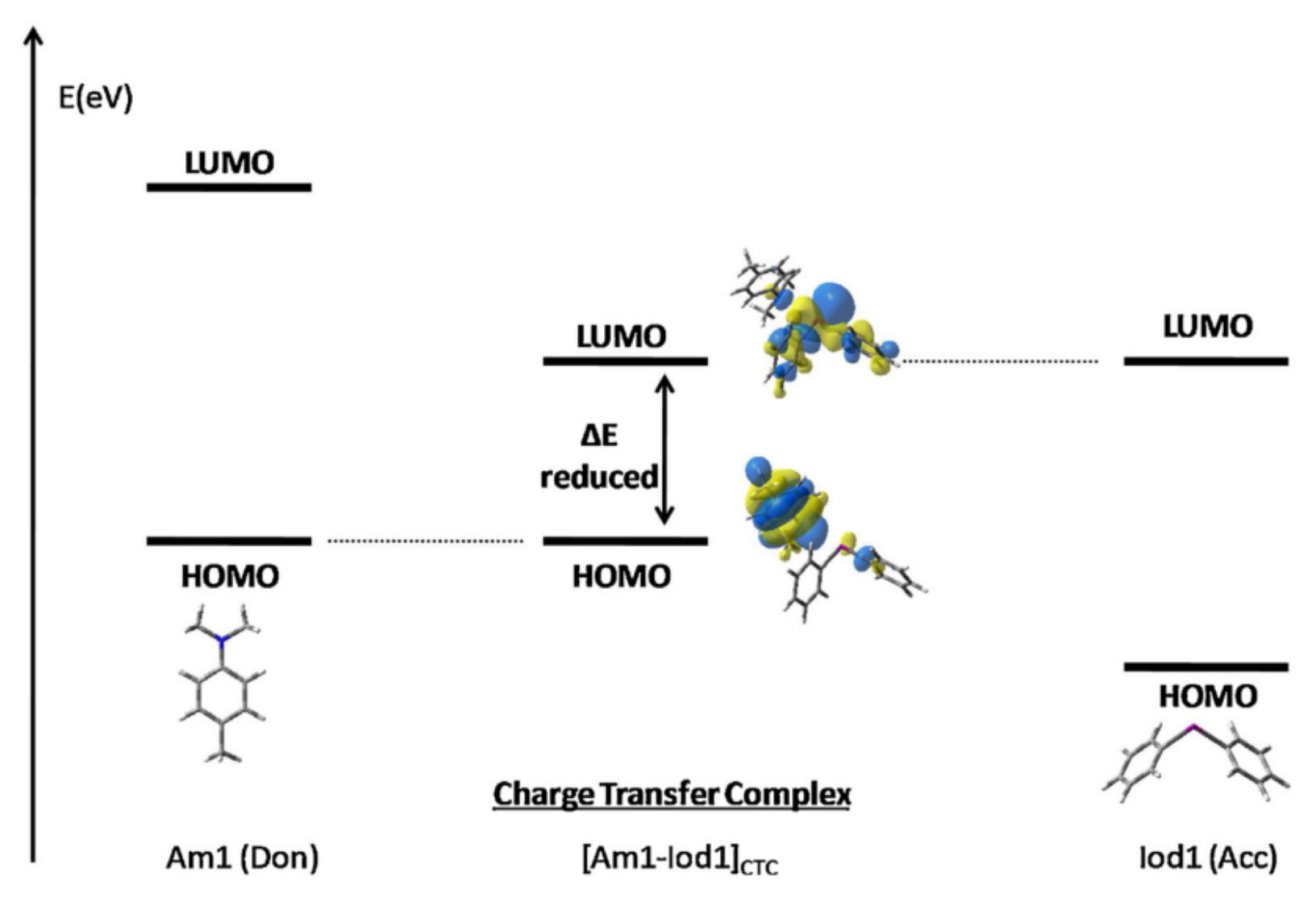
Simplified diagram of electron donor and electron acceptor interactions and frontier molecular orbitals of CTCs [95].

**Figure 5:**
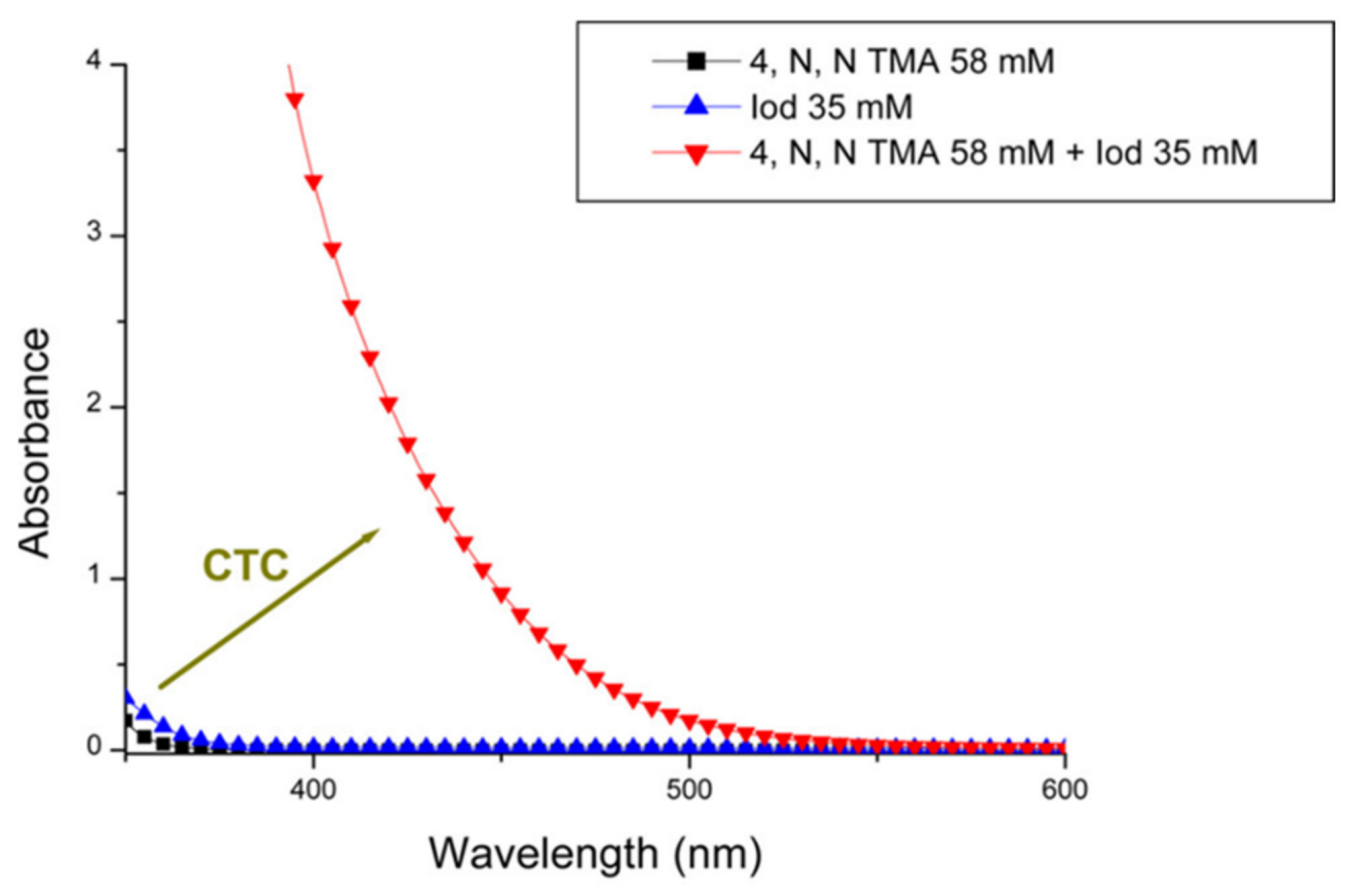
UV−vis absorption spectra of 58 mM 4,N,N TMA (black), 35 mM Iod (blue), and 58 mM 4,N,N TMA + 35 mM Iod mixed (red) in DCM [94].

The formation of CTC results in a certain color change in solution. For example the amine donor (4, N, N TMA) and acceptor (Iod) are colorless, but when they were mixed, a clear yellow can be observed (Figure 3) [94]. This is due to the contribution of the HOMO of amine and LUMO of Iod (Figure 4), which form a complex structure with a reduced energy gap compared to the isolated compound, leading to a red-shift in the absorption spectrum (Figure 5) [95].

**Scheme 18:**
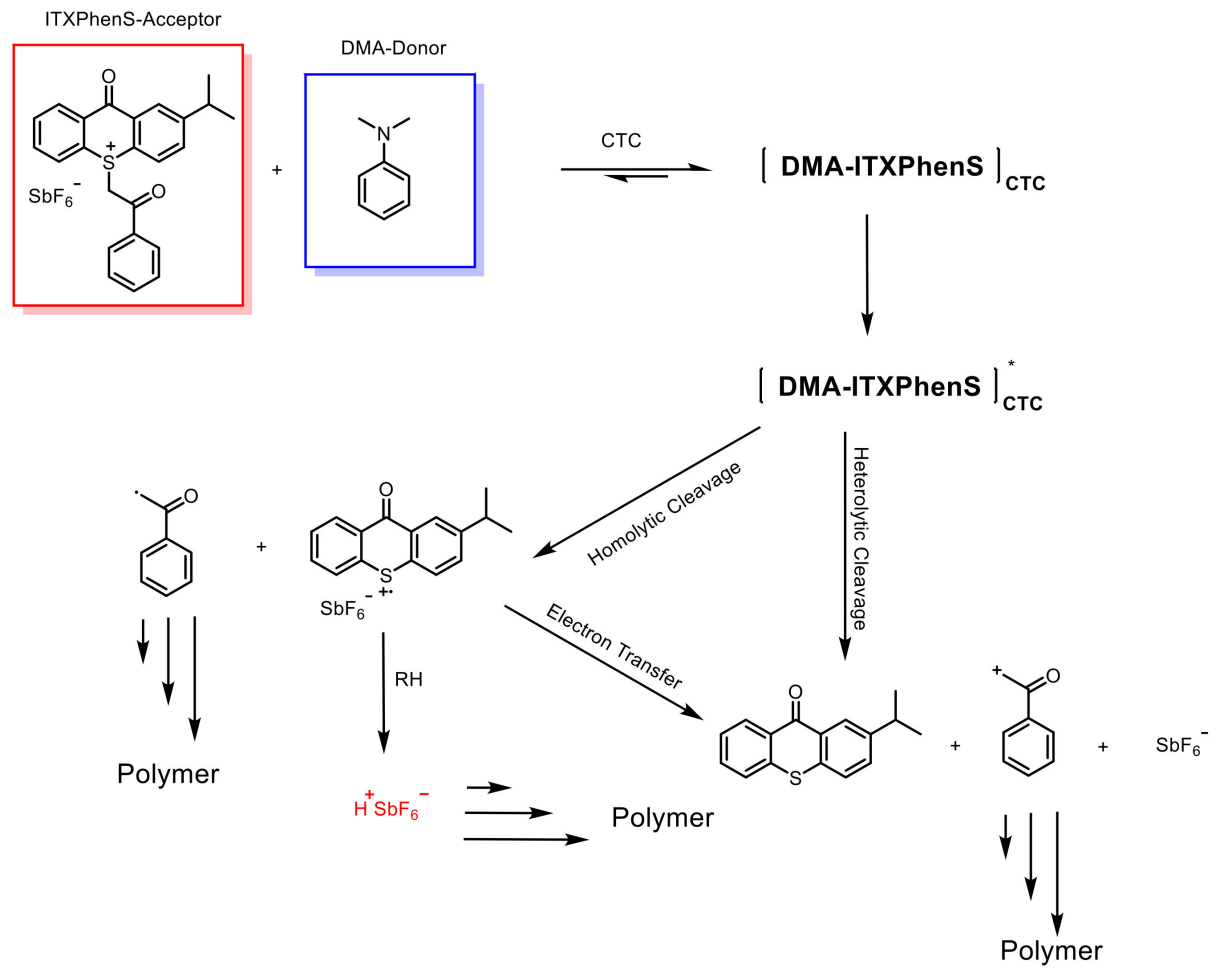
The mechanism of FRP and CP initiated by DMA-ITXPhenS under visible light [57]. Red color represent the active species that can initiate polymerization. * means that compound is in the excited state.

Yagci et al. [57] found that ITXPhenS and the donor N,N-dimethylaniline (DMA) could form a CTC with excellent absorption properties in the visible range. The range of the absorption spectrum red-shift can be controlled by the ratio of ITXPhenS and DMA. Under visible and natural sunlight, CTC can induce the generation of free radicals and ionic species through the heterolytic and/or homolytic cleavage of ITXPhenS, followed by an electron transfer reaction. The specific initiation mechanism is shown in Scheme 18.

**Figure 6:**
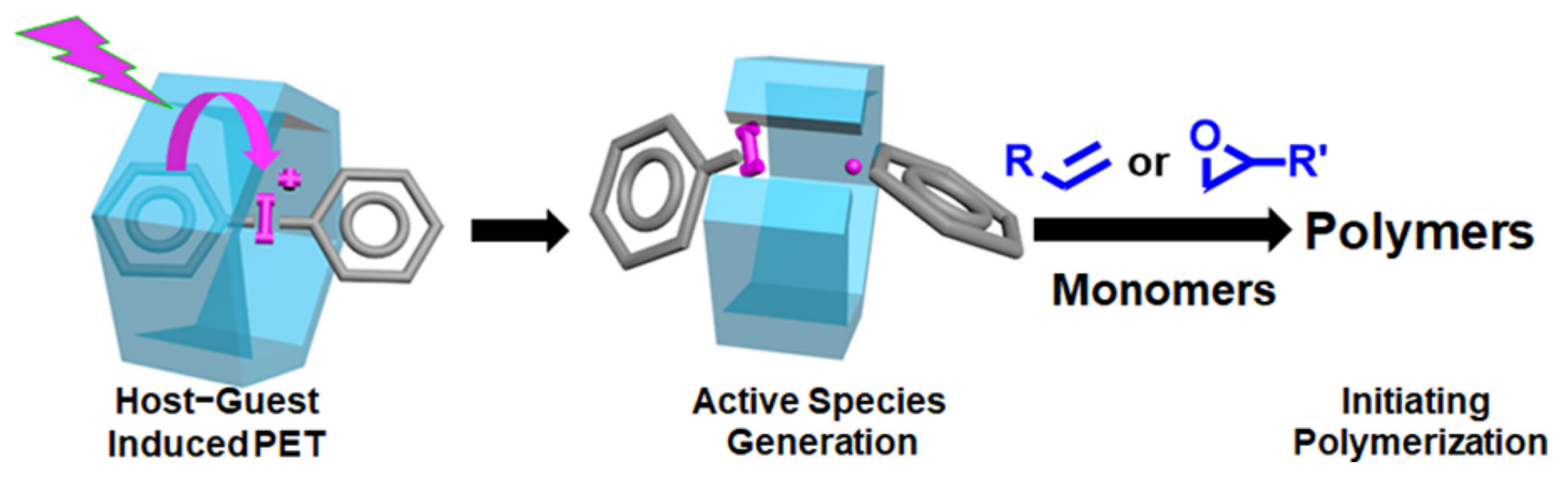
The mechanism of FRP and CP initiated by the supra-photoinitiator [96].

In 2021, Chang et al. [96] developed a host-guest complexation based on a macrocycle (pylarene, P6; prismatic arene, NP5) and diphenyliodonium salt (Iod), and prepared two novel supramolecular photoinitiators (ultraphotoinitiator). CTC can be formed between the host and the guest. Under light irradiation, the macrocyclic P6 or NP5 can provide electrons to the guest molecule Iod, and the Iod generates highly active free radicals and cationic fragments to achieve efficient polymerization (Figure 6). Electron transfer between the macrocycle and iodine is non-diffusion controlled, endowing a higher rate of photopolymerization and final conversion of the epoxy resin compared with commercial activators. In addition, the host-guest complexation of NP5 extends the onset wavelength of Iod from ultra-short ultraviolet to near ultraviolet, which can better match the environment-friendly LED light source. It is expected that the supra-photoinitiator may open up a new avenue for designing new photoinitiators with high performance.

### 3.2. Photosensitization by Electron Transfer in Exciplex

**Scheme 19:**
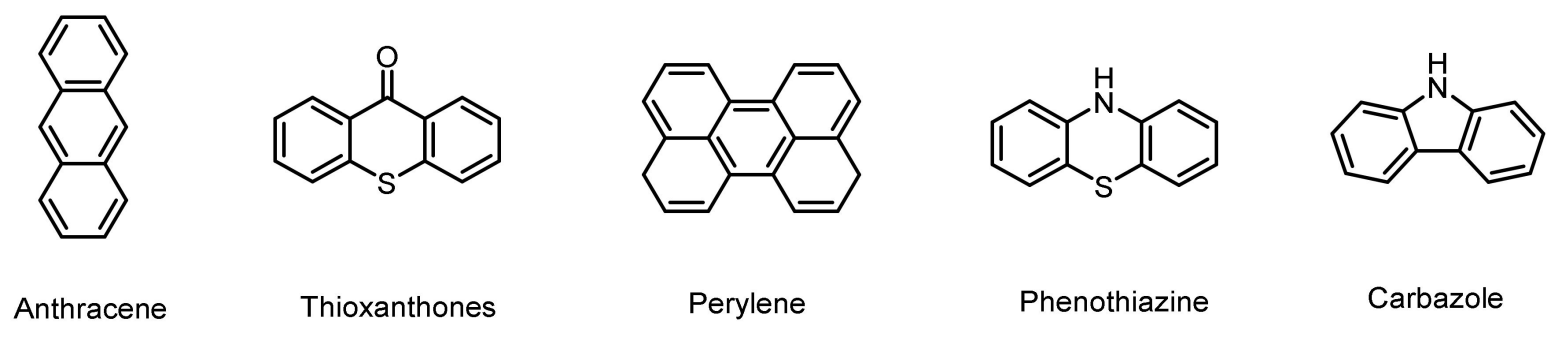
Chemical structures of several common photosensitive groups.

**Scheme 20:**
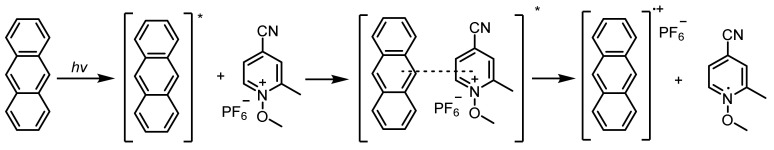
Photoinduced electron transfer reactions in the exciplex for CP of photosensitization [86]. * means that compound is in the excited state.

The photosensitizers are suitable for CP in the longer wavelength range. Many aromatic compounds such as anthracene [97,98,99], thioxanthones [100,101,102,103], perylene [104,105,106], phenothiazine [107,108,109,110] and carbazole [111,112,113,114,115] act as photosensitizers (Scheme 19). Under irradiation, the photosensitizer is excited to form a complex with the ground state onium salt, and the excited state photosensitizer transfers electrons to the onium salt to generate unstable free radicals and radical cations, thereby triggering subsequent CP (Scheme 20). The addition of photosensitizers greatly expanded the spectral range of CP. The successful polymerization the free energy change for the electron transfer (ΔG_et_), calculated according to Rehm-Weller equation, should be negative [116,117]:

$$\Delta G_{\mathrm{et}}=F[E_{1/2}^{ox}(\mathrm{PS})-{\;E}_{1/2}^{red}(\mathrm{On}+)]-E*+C$$

where F is the Faraday constant, and E* is the excitation energy of the sensitizer (singlet or triplet). Where $E_{1/2}^{ox}$ (V) and $E_{1/2}^{red}$ (V) are the electrochemical oxidation half-wave potential and reduction half-wave potential of PS salt and onium salt, respectively. C is the Coulomb term, which is often ignored due to the reaction corresponding to charge transfer.

Thanks to their photoconductive properties and facile oxidation, carbazoles are widely used in the design of organic materials and PIs [118]. In a recent study by Zhu et al. [119], N-methylpyrrole was also found to be a very effective molecule for the design of functional PIs. Soon after, the authors also designed bibenzylidene acetone, again using N-methylpyrrole as the electron-donating group, and concluded that the six-membered ring of the ketone part was not favorable for the photochemical reactivity of pyrrolyl enone dyes for photoinitiated applications [120]. Here, the authors [114] considered the combination of chalcone and N-methylpyrrole to synthesize a versatile and efficient PI that can be used for both FRP and CP, and subsequently they introduced a novel synthetic route to obtain a bifunctional pyrrole-carbazole-based photoinitiator (ECMO) by a one-step reaction. Due to its excellent absorption and hydrogen supply capability at 405 nm, ECMO can initiate radical polymerization under visible light irradiation to form colorless photopolymers. Notably, ECMO is able to further act as a sensitizer for triarylthiolium salts, significantly extending the absorption window of cesium salts into the visible spectral region. The ECMO-sensitized triarylthiolium salts exhibit excellent curing performance in cationic polymerization (e.g., higher terminal monomer conversion) compared to the conventional isopropylthioanthrone/hexafluorophosphate triarylthiolium salt system.

### 3.3. Free Radical Promotion

About forty years ago, Ledwith discovered free radical promoted cationic polymerization (FRPCP) [86]. Following this discovery, researchers have developed a number of FRPCP systems based on the difference between the reaction mechanism of free radicals and onium salts. FRPCP can proceed via two distinct mechanisms: (i) addition fragmentation and (ii) oxidation of electron donor radicals.

#### 3.3.1. Addition-Fragmentation Mechanism

**Chart 1:**
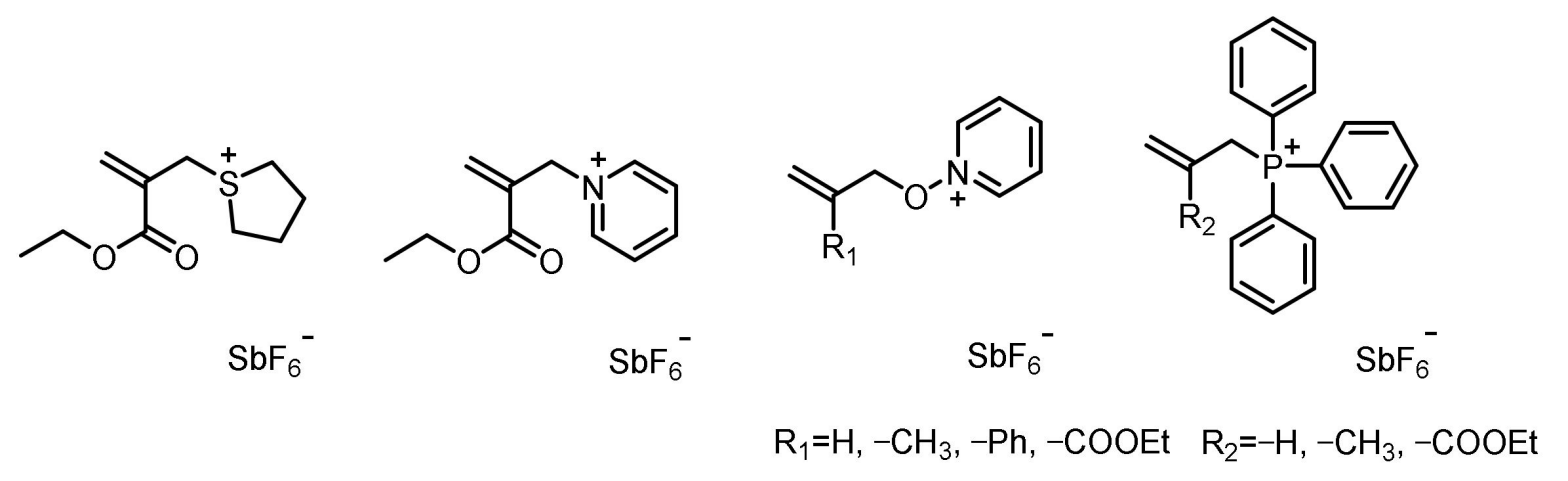
The chemical structure of allylonium salts used as addition fragmentation agents [121,122,123,124,125,126].

**Scheme 21:**
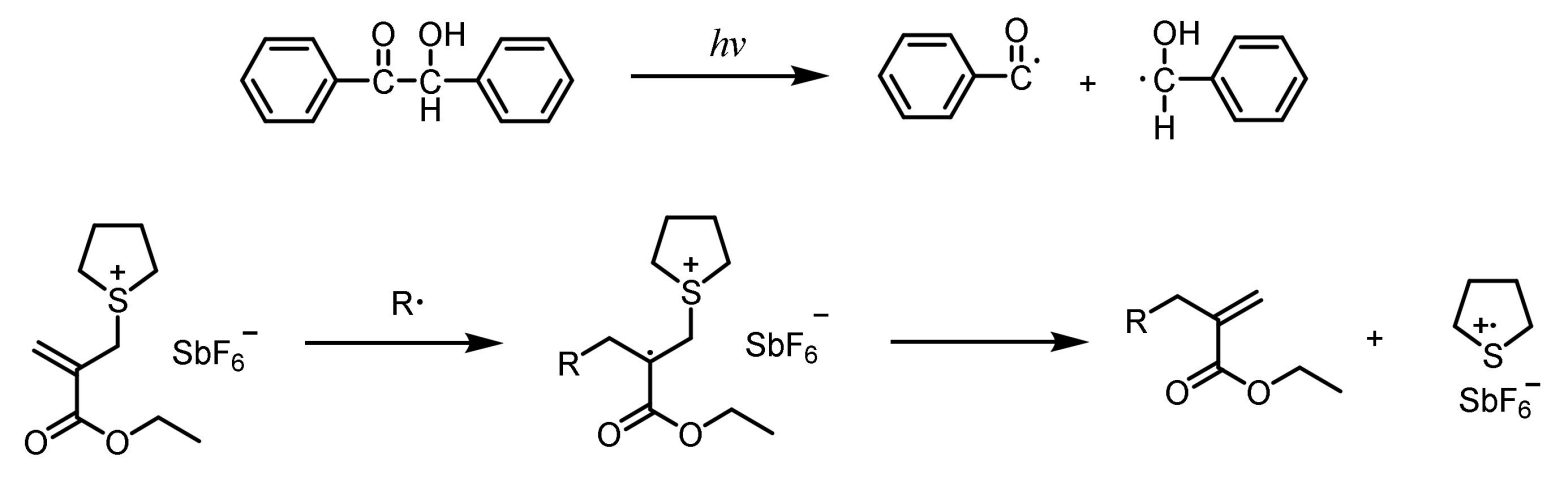
Photoinduced generation of reactive radical cations by addition fragmentation mechanism [127].

This concept is based on the specially designed allylic salts of the structures presented in Chart 1. These salts in conjunction with conventional free radical initiators were shown to initiate CP [121,122,123,124,125,126]. Both thermal and photo initiators can be used. The general reactions involved are presented in Scheme 20 for the 2,2-dimethoxy 2-phenyl acetophenone photoinitiator and allylic sulfonium salt combination. Photochemically formed radicals undergo addition reactions with allyl group to produce unstable radical intermediates that fragment to produce radical cationic active centers capable of initiating CP reaction. (Scheme 21).

**Scheme 22:**
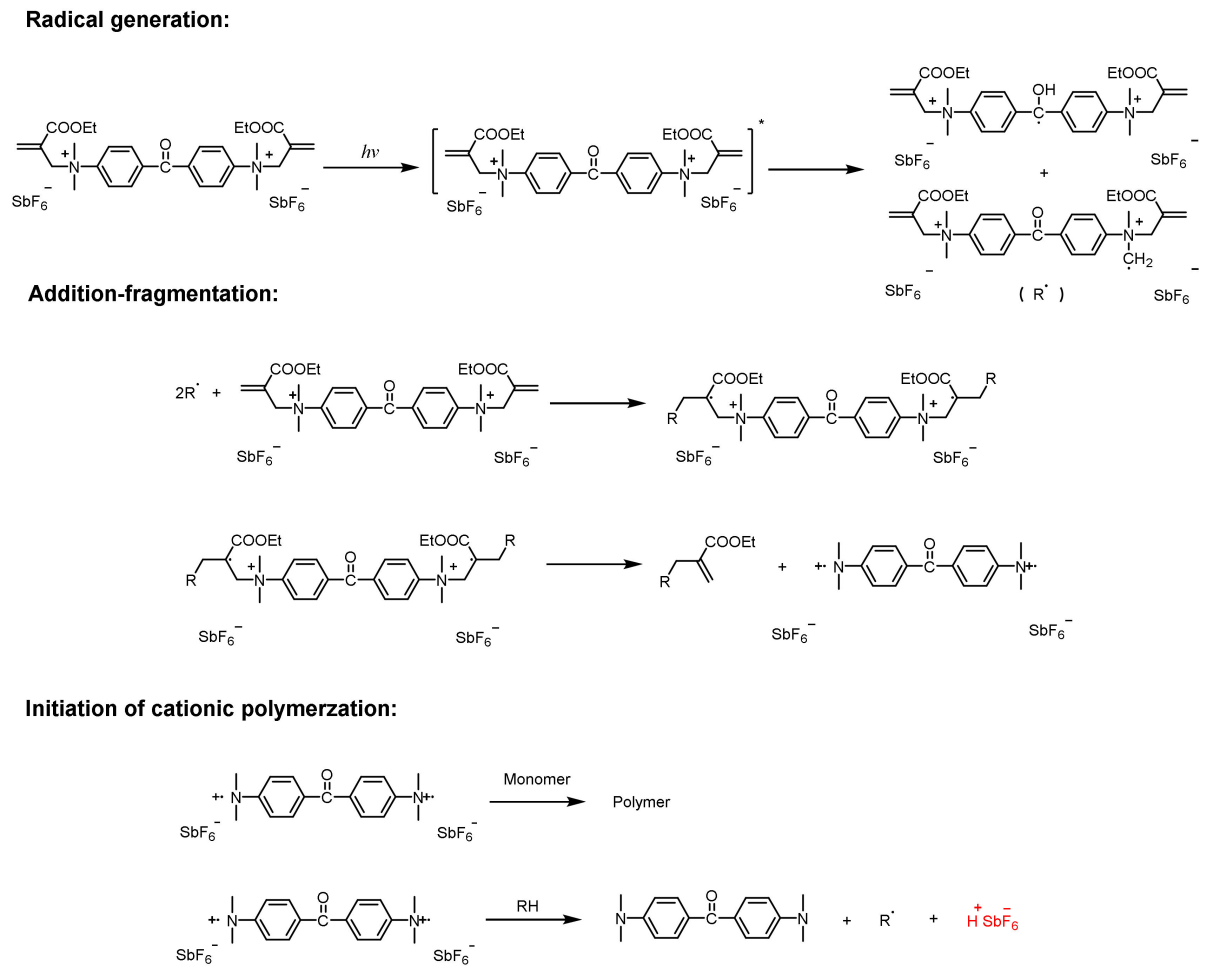
Mechanism of initiation by using allylammonium addition fragmentation agent containing benzophenone moieties [127]. Red color represent the active species that can initiate polymerization. * means that compound is in the excited state.

The mobility of the photosensitizer (free radical PI) molecule in the system affects the photopolymerization. As the viscosity of the system increases, the diffusion of photosensitizer to onium salt molecules becomes difficult, and the reaction between active radicals and onium salt molecules decreases. To overcome these limitations, allyl onium salts that also contained photoactive groups in the same molecule were synthesized [121,122,123]. The PIs does not need to supplement the free radical source, and the light-induced benzophenone unit undergoes a hydrogen abstraction reaction in which the generated radicals participate in addition and cleavage reactions, resulting in an active substance capable of initiating CP (Scheme 22).

#### 3.3.2. Oxidation of Free Radicals

**Scheme 23:**
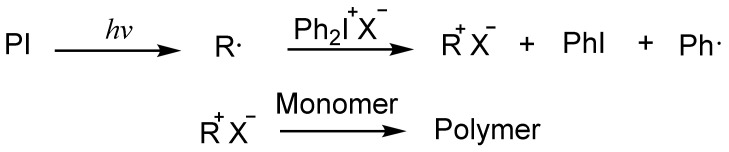
General mechanism of photoinduced free radical promoted cationic polymerization [137].

In 1978, Ledwith first proposed the oxidation of electron donor free radicals to promote CP [127]. The radicals generated by Type I or Type II radical PIs upon irradiation are oxidized by onium salts to form active cations (Scheme 23) [127,128,129,130,131,132,133,134,135]. Electron donor free radicals can not only be generated photochemically, but also by thermal means and high energy rays. Among them, the photochemical route benefits the advantage of being conducted at low temperature. Theoretically, the efficiency of the redox system reaction between onium salts and free radicals can be evaluated by the following formula [27,136]:

$$\Delta G=F[E_{1/2}^{ox}(R\cdot )-E_{1/2}^{red}({\mathrm{On}}^{+})]$$

where F is Faraday’s constant. $E_{1/2}^{ox}$ (R·) − $E_{1/2}^{red}$ (On^+^) are the half-wave oxidation and reduction potentials of radicals and onium salts, respectively.

As an oxidizing agent, the efficiency of the onium salt is positively correlated with its electron nucleophilicity. The stronger the electron nucleophilicity, the stronger its reduction potential $E_{1/2}^{red}$ (On+) and the stronger its ability to oxidize free radicals. However, there is an obvious defect in this formula. We can easily obtain the reduction potential of onium salts. However, since free radicals exist as intermediate substances and cannot be stable in the system for a long time for measurement, it is difficult to obtain their oxidation potentials.

**Scheme 24:**
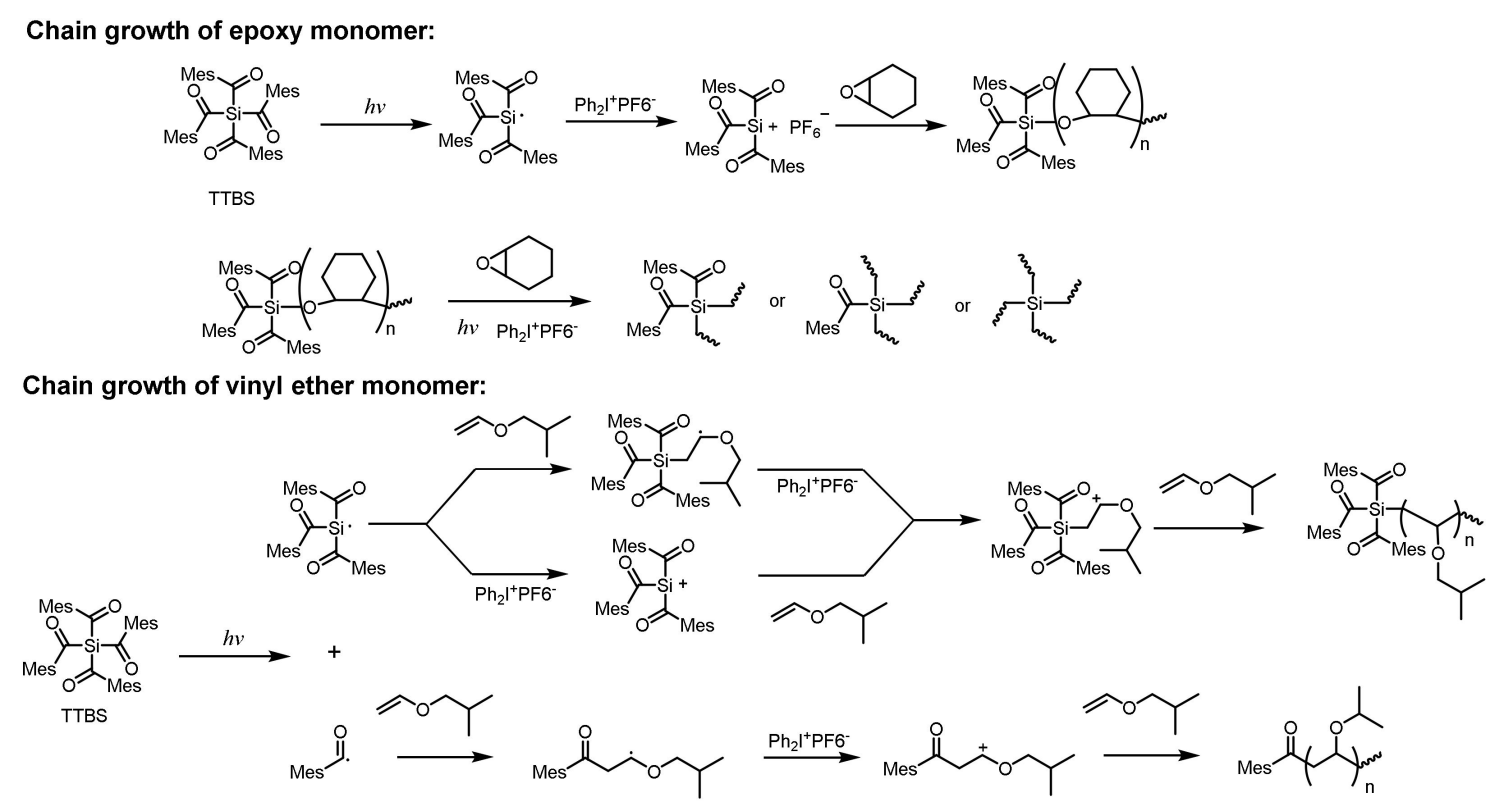
Visible photoinitiated polymerization of cyclohexene oxide and isobutyl vinyl ether using TTBS -Ph_2_I^+^PF_6_^−^ photoredox pair [138].

Yagci et al. [137] designed a novel visible-light CPIS consisting of a silicon-based PI and an iodonium salt. The important features of this initiating system are (i) the generation of silyl radicals by cleavage using visible light, and (ii) the electron transfer reaction of the resulting radicals with diaryliodonium salts to generate the corresponding cations to initiate CP. Acyl radicals formed by photoinitiated systems can generate reactive cations through addition reactions and oxidation processes, and the article also demonstrates the continuous photolytic properties of tetrakis(2,4,6-trimethylbenzoyl)silane (TTBS) (Scheme 24).

In 2018, Yagci et al. [138] demonstrated for the first time that organotelluric compounds together with iodonium salts are efficient visible-light photoinitiators for radical-promoted cationic polymerization of various monomers. Cationic photopolymerization was successfully achieved by the formation of carbon-centered radicals under visible light irradiation in a manner similar to that of living radical polymerization. In recent years, Zhu et al. [139,140] reported a photoinduced radical-promoted cationic reversible addition-fragmentation chain transfer polymerization of vinyl ether, which can construct ‘living’ objects at a relatively fast construction speed (12.99 cm/h) through a commercial DLP 3D printer.

## 4. Conclusions and Outlook

CP has attracted extensive attention in both academic and industrial areas as it offers advantages such as insensitivity to oxygen, low shrinkage, strong temporal and spatial control during of the polymerization process and enables the preparation of various polymer structures in environmentally friendly conditions. In recent decades, there have been tremendous efforts focused on the development of new photoinitiating systems that can be activated at higher wavelengths. Moreover, CPISs have been combined with FRP in a concurrent manner for the preparation of hybrid materials and IPN, as many systems generate both cationic and radical species. A large number of CPIs that can be excited in the near-UV, visible light and even NIR regions have been developed. It is expected that more intensive research interest will focus on the development of new PIs having desired absorption characteristics, prepared by easy synthetic methodologies, matching well with the energy conservation and green chemistry principals.

## Figures and Tables

**Table 1:** Photophysical properties of different iodonium salts.

Structure	*λ*_max_ (nm)	*ε* (L·mol^−1^·cm^−1^)	Ref.
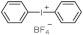	227	17,800	[27]
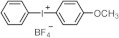	246	15,400	[27]
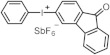	267 298 380 394	4200 8500 430 400	[28]
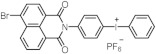	340 360	/ /	[29]
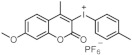	349	22,704	[15]
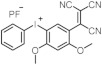	422	8700	[30]
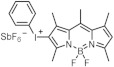	475	30,869	[31]
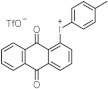	336	5200	[32]
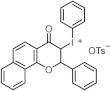	344	6730	[32]

**Table 2:** Photophysical properties of different triarylsulfonium salts.

Structure	*λ*_max_ (nm)	*ε* (L·mol^−1^·cm^−1^)	Ref.
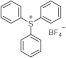	227	21,000	[40]
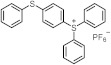	230 300	24,330 19,500	[40]
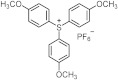	255 280	21,700 10,100	[27]

**Table 3:** Photophysical properties of different aryl-alkylsulfonium salts.

Structure	*λ*_max_ (nm)	*ε* (L·mol^−1^·cm^−1^)	Ref.
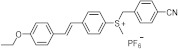	347	36,100	[49]
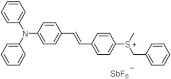	397	34,400	[48]
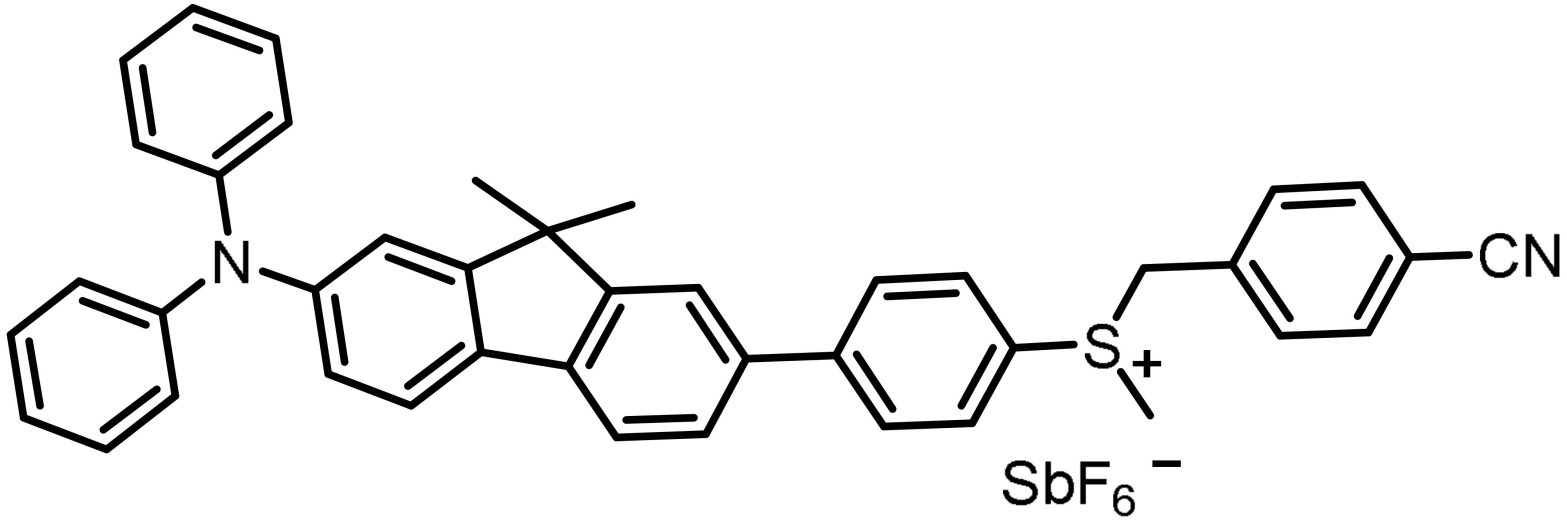	378	39,400	[47]
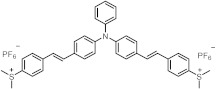	413	37,000	[46]
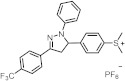	372	16,700	[44]

**Table 4:** Photophysical properties of different phenacylsulfonium salts.

Structure	*λ*_max_ (nm)	*ε*(L·mol^−1^·cm^−1^)	Ref.
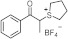	255 315	11,475 2063	[55]
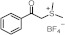	250 280	10,890 4507	[51]
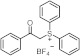	~250	/	[52]
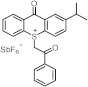	~260 ~380	/ /	[57]
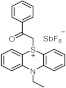	514	5151	[53]
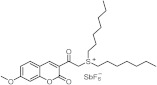	372	18,568	[54]

**Table 5:** Photophysical properties of different phosphonium salts.

Structure	*λ*_max_ (nm)	*ε* (L·mol^−1^·cm^−1^)	Ref.
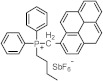	280 350	38,000 40,000	[60]
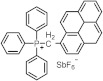	280 350	30,800 31,200	[60]
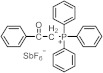	257	32,000	[59]

**Table 7:** Photophysical properties of different ammonium salts.

Structure	*λ*_max_ (nm)	*ε* (L·mol^−1^·cm^−1^)	Ref.
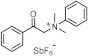	249 279	/	[67]
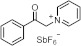	249 279 438	/	[69]
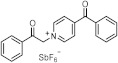	252 507	/	[69]
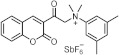	~320 ~360	/	[71]

**Table 8:** Photophysical properties of different ferrocenium salts.

Structure	*λ*_max_ (nm)	*ε* (L·mol^−1^·cm^−1^)	Ref.
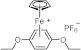	243 397 462	14,900 136 78	[76]
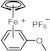	242 389 465	14,700 136 72	[76]
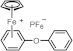	243 397 462	19,400 140 75	[76]
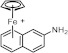	213 242 376 546	21,600 20,100 2380 967	[77]
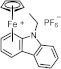	219 259 352 429	23,700 28,700 2320 187	[78]

**Table 9:** Photophysical properties of three new thiophenoxime containing sulfonic acid compounds [85].

Structure	*λ*_max_ (nm)	*ε* (L·mol^−1^·cm^−1^)
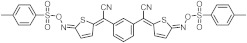	425	18,900
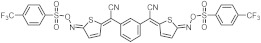	426	15,900
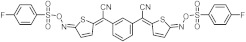	423	13,400

## Data Availability

No new data were created.
